# Modeled Health and Economic Burden of Frailty and Falls Among Adults With HIV

**DOI:** 10.1001/jamanetworkopen.2025.54809

**Published:** 2026-01-21

**Authors:** Karen C. Smith, Cathryn Brown, Emily P. Hyle, Reyhaneh Zafarnejad, Todd T. Brown, Kenneth A. Freedberg, Kristine M. Erlandson, Elena Losina

**Affiliations:** 1 Orthopedic and Arthritis Center for Outcomes Research (OrACORe), Department of Orthopedic Surgery, Brigham and Women’s Hospital, Boston, Massachusetts; 2 Harvard Medical School, Boston, Massachusetts; 3 Medical Practice Evaluation Center (MPEC), Massachusetts General Hospital, Boston; 4 Division of Infectious Diseases, Department of Medicine, Massachusetts General Hospital, Boston; 5 Division of Endocrinology, Diabetes, and Metabolism, Johns Hopkins University School of Medicine, Baltimore, Maryland; 6 Division of General Internal Medicine, Department of Medicine, Massachusetts General Hospital, Boston; 7 Department of Medicine, Division of Infectious Diseases, University of Colorado–Anschutz Medical Campus, Aurora

## Abstract

**Importance:**

People with HIV experience higher rates of frailty and falls than age-matched people without HIV.

**Objective:**

To estimate the life-years lost, quality-adjusted life-years (QALYs) lost, and costs attributable to prefrailty, frailty, and falls among people with HIV and viral suppression in the United States.

**Design, Setting, and Participants:**

This decision analytic modeling study used the Frailty Policy Model, a microsimulation model, to project lifetime health and cost outcomes associated with frailty and falls among people with HIV in the United States. The model simulated individuals representing people with HIV and viral suppression in the United States aged 40 years and older in 2022, and results were scaled to the estimated population size of 521 994 individuals. Simulation model parameters were drawn from the Advancing Clinical Therapeutics Globally (ACTG) A5322 Study, the Multicenter AIDS Cohort Study (MACS)/Women’s Interagency HIV Study (WIHS) Combined Cohort Study, and published literature. Data analysis was conducted from November 2023 to October 2025.

**Exposures:**

Prefrailty, frailty, and falls.

**Main Outcomes and Measures:**

Life-years lost, QALYs lost, and costs attributable to prefrailty, frailty, and falls.

**Results:**

The simulated individuals representing people with HIV and viral suppression had a mean (SD) age of 56 (10) years; 25% were female; 41% had prefrailty, and 7% had frailty. The model projected that the simulated individuals would have a remaining life expectancy of 20.3 (95% uncertainty interval [UI], 19.7-20.8) years, with a mean of 12.0 (95% UI, 11.2-12.8) years with prefrailty or frailty and 10.1 (95% UI, 8.2-12.1) falls per person. Scaled to the population level, the model projected that there would be 31 000 (95% UI, 16 000-57 000) life-years lost, 214 000 (95% UI, 130 000-292 000) QALYs lost, and $5.0 (95% UI, $3.2-$7.2) billion in lifetime costs attributable to prefrailty. There would be 1 352 000 (95% UI, 84 000-3 336 000) life-years lost, 1 091 000 (95% UI, 209 000-2 500 000) QALYs lost, and $8.8 (95% UI, $4.7-$14.2) billion in lifetime costs attributable to frailty. There would be 183 000 (95% UI, 120 000-266 000) life-years lost, 141 000 (95% UI, 94 000-198 000) QALYs lost, and $3.4 (95% UI, $2.2-$4.8) billion attributable to falls.

**Conclusions and Relevance:**

In this decision analytic modeling study of frailty and falls among people with HIV and viral suppression, the lifetime QALYs lost and costs attributable to frailty and falls were substantial. These findings highlight the potential clinical and economic benefits that could result from interventions to identify, prevent, and treat frailty and falls among people with HIV.

## Key Points

**Question:**

What are the anticipated lifetime health losses and costs attributable to frailty and falls among people with HIV (PWH) and viral suppression in the United States?

**Findings:**

In this decision analytic model with results scaled to an estimated population size of 521 994 PWH, there would be a projected 214 000 quality-adjusted life-years (QALYs) lost and $5.0 billion in lifetime costs attributable to prefrailty among PWH. There would be a projected 1 091 000 QALYs lost and $8.8 billion attributable to frailty and a projected 141 000 QALYs lost and $3.4 billion attributable to falls.

**Meaning:**

The findings of this study suggest that the anticipated clinical and economic burden of frailty and falls among aging PWH in the United States could be substantial.

## Teaser

This decision analytic model examines the health and cost outcomes associated with frailty among adults aged 40 years and older in the United States who have HIV and viral suppression.

## Introduction

The life expectancy of people with HIV (PWH) and viral suppression in the United States is approaching that of people without HIV.^1,2^ However, PWH experience higher rates of age-related comorbidities, such as cardiovascular disease, cancer, liver disease, and fractures, at earlier ages and with greater severity than people without HIV.^1,3,4,5,6,7^ This increased comorbidity burden is likely due to chronic inflammation, immune activation, effects of antiretroviral therapies, and disadvantageous social determinants of health and risk factors, such as tobacco use.^3,5,8,9^ For example, higher tobacco use contributes to increased cancer burden,^10^ while antiretroviral therapies may decrease bone mineral density,^11^ leading to greater fracture risk.

Prefrailty and frailty are among the age-related syndromes that affect PWH at higher rates and younger ages compared with people without HIV.^12,13,14^ Frailty refers to a state of decreased physiologic reserve in which an individual has increased vulnerability to adverse events and stressors.^15^ Frailty may occur at higher rates among PWH due to multiple reasons from biologic mechanisms (dysregulated inflammation, immune activation, mitochondrial dysfunction), to increased comorbidity burden and socioeconomic burdens.^5,16,17^ Among PWH, adults with frailty report worse quality of life and have higher rates of hospitalization than adults without frailty.^18,19^ Prefrailty and frailty are also risk factors for falls and fall-related injuries, which occur frequently among PWH, even at younger ages.^20,21,22,23,24^

Given the increased risk of frailty and falls among PWH, our objective was to use disease simulation modeling informed by longitudinal studies of aging PWH to estimate health losses and lifetime costs attributable to prefrailty, frailty, and falls among PWH with viral suppression in the United States. Health-related behavior change, such as participation in frailty and fall prevention programs, is motivated by perceived susceptibility, perceived severity, and perceived benefits.^25,26^ Our estimates of health losses and financial costs provide evidence regarding the susceptibility to and severity of frailty and falls, as well as the potential benefits of prevention and treatment. These estimates may therefore be used by patients, clinicians, and policymakers to support engagement in frailty and fall prevention. The results may also motivate and inform future work on the value of frailty and fall prevention and treatment for PWH.

## Methods

### Analytic Overview

We developed the Frailty Policy Model, a simulation model of frailty and fall-related injuries among PWH. The model was informed by longitudinal data from the Advancing Clinical Therapeutics Globally for HIV/AIDS and Other Infections (ACTG) A5322 Study, the Multicenter AIDS Cohort Study (MACS)/Women’s Interagency HIV Study (WIHS) Combined Cohort Study (MWCCS), and published literature (eTable 1 in Supplement 1).^27,28,29,30,31,32,33,34,35,36,37^ Using the model, we projected lifetime health and cost outcomes for PWH in the United States aged 40 years and older in 2022. The goal of the Frailty Policy Model is to address health care policy and delivery questions related to the prevention and treatment of frailty and falls among PWH. This analysis provides estimates of the burden of frailty and falls among PWH and will serve as a foundation for future policy analyses investigating the value of frailty and fall prevention.

The methods section is organized as follows: we describe (1) the simulated cohort, (2) the model structure and outcomes, (3) data inputs, (4) uncertainty analyses, and (5) validation analyses. This research was determined to be exempt by the Brigham and Women's Hospital Institutional Review Board. The reporting follows the Consolidated Health Economic Evaluation Reporting Standards (CHEERS) reporting guideline. We used R version 4.4.2 (R Project for Statistical Computing) to develop the simulation model and conduct statistical analyses.

### Cohort Characteristics

We simulated PWH with viral suppression aged 40 years and older in the United States using age and sex distributions reported by the CDC.^27,28^ For population-level estimates, we used CDC data to estimate that 521 994 PWH with viral suppression were aged 40 years and older in 2022 in the United States, and we scaled results to this population size (eTable 2 in Supplement 1). To assign characteristics to each simulated individual, we used distributions estimated from the ACTG A5322 study.^29^ The mean (SD) age was 56 (10) years, 41% of the cohort had prefrailty, and 7% had frailty (Table 1; eTable 3 in Supplement 1).

**Table 1.** Cohort Characteristics and Model Parameters for Simulation of Prefrailty, Frailty, and Falls Among People With HIV and Viral Suppression in the United States

Parameter	Point estimate	Distribution^a^	Source
**Cohort characteristics**			
Population size, No.	521 994	NA	Derived from CDC HIV surveillance reports^27,28^
Age, mean (SD), y	56 (10)	NA	
Female, %	25	NA	
Prefrail, %	41	Bootstrapped	Derived from ACTG A5322^29^
Frail, %	7	Bootstrapped	
Transition probabilities, %			
Age <50 y			Derived from ACTG A5322^29^
Nonfrail to prefrail	27	Poisson (mean, 304)	
Prefrail to nonfrail	33	Poisson (mean, 289)	
Prefrail to frail	8	Poisson (mean, 57)	
Frail to prefrail	44	Poisson (mean, 48)	
Age 50-64 y			
Nonfrail to prefrail	22	Poisson (mean, 488)	
Prefrail to nonfrail	23	Poisson (mean, 432)	
Prefrail to frail	10	Poisson (mean, 173)	
Frail to prefrail	39	Poisson (mean, 154)	
Age ≥65 y			
Nonfrail to prefrail	25	Poisson (mean, 79)	
Prefrail to nonfrail	13	Poisson (mean, 51)	
Prefrail to frail	15	Poisson (mean, 57)	
Frail to prefrail	39	Poisson (mean, 35)	
Falls	Stratified by individual characteristics^b^	Multivariate normal^c^	
Injury given a fall, female			
Nonfracture injury	23.0	Multivariate normal^c^	
Fracture	8.6	Multivariate normal^c^	
Injury given a fall, male			
Nonfracture injury	15.5	Multivariate normal^c^	
Fracture	5.8	Multivariate normal^c^	
Fracture site distribution, %			
Female			Derived from MWCCS^30^
Hip	4	Dirichlet (4, 3, 23, 64)	
Spine	3		
Wrist and arm	24		
Other	68		
Male			
Hip	2	Dirichlet (3, 5, 24, 97)	
Spine	4		
Wrist and arm	19		
Other	75		
Mortality parameters			
All-cause mortality	Age and sex stratified^d^	NA	CEPAC model estimates^31^
Frailty mortality hazard ratio	3.19	logNormal (mean [SD], 1.16 [0.58])	Verheij et al,^32^ 2020
Hip fracture mortality hazard ratio	6.28	logNormal (mean [SD], 1.84 [0.14])	Tosteson et al,^33^ 2007
**Frailty, injury, and fracture health-related quality-of-life decrements**			
Prefrailty, male			Derived from MWCCS^30^
No chronic pain	0.02	Multivariate normal^c^	
Chronic pain	0.05	Multivariate normal^c^	
Prefrailty, female			
No chronic pain	0.04	Multivariate normal^c^	
Chronic pain	0.07	Multivariate normal^c^	
Frailty, male			
No chronic pain	0.06	Multivariate normal^c^	
Chronic pain	0.12	Multivariate normal^c^	
Frailty, female			
No chronic pain	0.09	Multivariate normal^c^	
Chronic pain	0.15	Multivariate normal^c^	
Nonfracture injury			Derived from Raich et al,^34^ 2023
Year 1	0.013	Gamma (mean [SD], 0.013 [0.002])	
Year 2	0.006	Gamma (mean [SD], 0.006 [0.003])	
Hip fracture			
Year 1	0.067	Gamma (mean [SD], 0.067 [0.013])	
Year 2	0.020	Gamma (mean [SD], 0.020 [0.005])	
Spine fracture			
Year 1	0.047	Gamma (mean [SD], 0.047 [0.010])	
Year 2	0.057	Gamma (mean [SD], 0.057 [0.011])	
Wrist or arm			
Year 1	0.032	Gamma (mean [SD], 0.032 [0.009])	
Year 2	0.023	Gamma (mean [SD], 0.023 [0.009])	
Other			
Year 1	0.037	Gamma (mean [SD], 0.037 [0.004])	
Year 2	0.020	Gamma (mean [SD], 0.020 [0.005])	
**Costs, 2023 US $**			
Prefrailty			Derived from Ensrud et al,^35^ 2023
Female	3400	Gamma (mean [SD], 3400 [800])	
Male	0	NA	
Frailty			
Female	9200	Gamma (mean [SD], 9200 [1200])	
Male	6600	Gamma (mean [SD], 6600 [1600])	
Nonfracture Injury	3300	Gamma (mean [SD], 3300 [700])	Derived from Peterson et al,^36^ 2021
Hip fracture	49 800	Gamma (mean [SD], 49 800 [10 000])	Derived from Hansen et al,^37^ 2021
Spine fracture	20 300	Gamma (mean [SD], 20 300 [4100])	
Wrist or arm fracture	12 600	Gamma (mean [SD], 12 600 [2500])	
Other fracture	14 300	Gamma (mean [SD], 14 300 [2900])	

Abbreviations: ACTG, Advancing Clinical Therapeutics Globally; CDC, US Centers for Disease Control and Prevention; CEPAC, Cost-effectiveness of Preventing AIDS Complications; MWCCS, MACS/WIHS Combined Cohort Study; NA, not applicable.

^a^ Lognormal and gamma distributions are shown with mean and standard deviation. Poisson distributions were used to draw rates, which were then converted to probabilities using follow-up time.

^b^ Stratified by individual characteristics (eTable 5 in Supplement 1).

^c^ Parameters with multivariate normal distributions were estimated from regression models. In each iteration of the analysis, coefficients were sampled from the regression coefficients and variance covariance matrix.

^d^ Age- and sex-stratified mortality rates are from projections of mortality among people with HIV on antiretroviral therapy from the CEPAC Model.

### Model Structure

At the beginning of the simulation, simulated individuals are assigned characteristics including prefrailty, frailty, comorbidities, smoking status, and prescription of opioids and psychotropic medications. We modeled opioids and psychotropics because they are associated with increased fall risk and functional impairment among PWH.^24,38,39,40^

**Figure 1.**
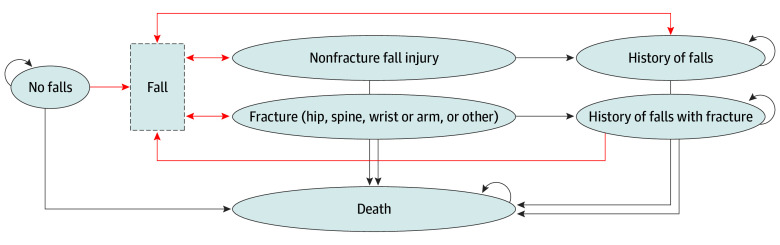
Model Structure The figure shows a model diagram describing the fall and fall-related injuries module of the simulation model. Ovals with solid borders indicate health states, and arrows indicate possible transitions between health states. Arrows in red indicate a transition between states that occurred due to a fall. Individual-level characteristics, such as frailty, are tracked for each individual as they move through these health states, and these characteristics affect the probabilities of transitioning between states.

Each year, simulated individuals face probabilities of changes in individual-level characteristics and transitions between health states, which include falls, fall-related nonfracture injuries, fall-related fractures (hip, spine, wrist or arm, and other), and death (Figure 1; eMethods in Supplement 1). Tracking individual-level characteristics occurs together with transitions between health states. For example, a simulated individual can experience frailty and falls in the same model cycle. Prior falls increase the risk of subsequent falls, and hip fractures and frailty increase mortality risk.^32,33,41^

Individual-level characteristics (ie, prefrailty, frailty, comorbidities, smoking status, and medications) affect cost and health outcomes by increasing fall risk. In addition, prefrailty and frailty contribute to cost and health outcomes independently of falls.

### Outcomes

We calculated life-years lost, quality-adjusted life-years (QALYs) lost, and costs attributable to prefrailty, frailty, or falls as the difference in outcomes between the status quo, which includes prevalent prefrailty, frailty, and falls, and 3 alternative scenarios. Each scenario removed either prefrailty, frailty, or falls. Prefrailty and frailty were removed from the simulation by setting the characteristics of prefrailty and frailty (eg, health-related quality-of-life weights, costs, and mortality risks) to those of nonfrailty. Falls were removed by setting the probability of falls to 0. Because our results are a description of disease burden, we report undiscounted outcomes in the base case and discounted outcomes in sensitivity analyses.

### Statistical Analysis

#### Data Inputs

##### Transition Probabilities

We used ACTG A5322 study data to estimate probabilities of changes in fall risk factors, including prefrailty, frailty, comorbidities, smoking, and prescription of opioids or psychotropics (eTable 4 in Supplement 1) as well as the probabilities of falls and fall-related injuries. Prefrailty and frailty were defined using the ACTG A5322 modified criteria of Fried and colleagues.^15^ Simulated individuals could experience improving or worsening frailty status each year of the simulation, and the probabilities that determine changes in frailty (Table 1) were stratified by age.

The annual probability of falls was calculated with a logistic regression that modeled falls as a function of age, sex, frailty status, comorbidities, medications, smoking status, and prior falls. Prefrailty and frailty increased the odds of falls by 1.7 and 2.8, respectively (eTable 5 in Supplement 1). Injury probabilities (Table 1) were based on ACTG A5322 participants’ self-report of whether a fall resulted in an injury requiring medical attention or a bone fracture. Fracture site was not reported in ACTG A5322, so we drew this estimate from the sex-stratified distribution of self-reported fall-related fractures in MWCCS.

##### Mortality

We used mortality estimates from the Cost-Effectiveness of Preventing AIDS Complications model.^31,42^ These estimates represent mortality among PWH taking antiretroviral therapy, accounting for the increased risk of mortality among PWH due to non-HIV–related factors.^42^ We adjusted estimates to represent mortality for PWH with nonfrailty, prefrailty, and frailty.^32^ Hip fractures also increased mortality.^33^

##### Health-Related Quality-of-Life

We modeled the health-related quality-of-life weight associated with each health state as a function of age, sex, comorbidities, and frailty status using self-reported health-related quality-of-life in the MWCCS (eTable 6 in Supplement 1). We derived decrements in health-related quality-of-life for nonfracture fall injuries and fall-related fractures from decrements reported in a study of QALY losses due to injuries.^34^ We included these decrements in the 2 years following an injury and limited to the first year only in sensitivity analyses.

##### Costs

All costs were adjusted to 2023 US dollars using the Personal Health Care index.^43^ The annual incremental costs of prefrailty and frailty were from a study of community-dwelling Medicare beneficiaries that reported incremental costs associated with frailty after adjusting for multimorbidity and chronic conditions.^35^ We estimated the cost of a nonfracture fall injury and fall-related fractures from published literature.^36,37,44^ Costs and health-related quality-of-life weights are shown in Table 1 and are further described in the eMethods in Supplement 1.

#### Uncertainty Analyses

We conducted deterministic sensitivity analyses varying key model parameters across plausible ranges (eTable 7 in Supplement 1). We also modeled 2 alternative scenarios. First, we compared the status quo for PWH with scenarios in which reductions in frailty and falls were estimated from studies of frailty and fall prevention programs. Second, we compared the status quo for PWH with scenarios in which PWH were assigned prefrailty prevalence, frailty prevalence, and fall risk based on estimates for people without HIV.

Our estimates are from probabilistic sensitivity analyses using 1000 parameter sets drawn from distributions in Table 1. We report results with the mean of the probabilistic analyses, and the uncertainty is reflected in 95% uncertainty intervals (UI).

#### Model Validation

We conducted internal model validations by simulating individuals based on the ACTG A5322 study and comparing simulated outcomes with observed outcomes over 5 years (eFigures 1-2 in Supplement 1). We also conducted external validations comparing model-projected frailty and fall incidence over 2 years with independent sources (eFigures 3-4 in Supplement 1).

## Results

### Status Quo Projections

**Figure 2.**
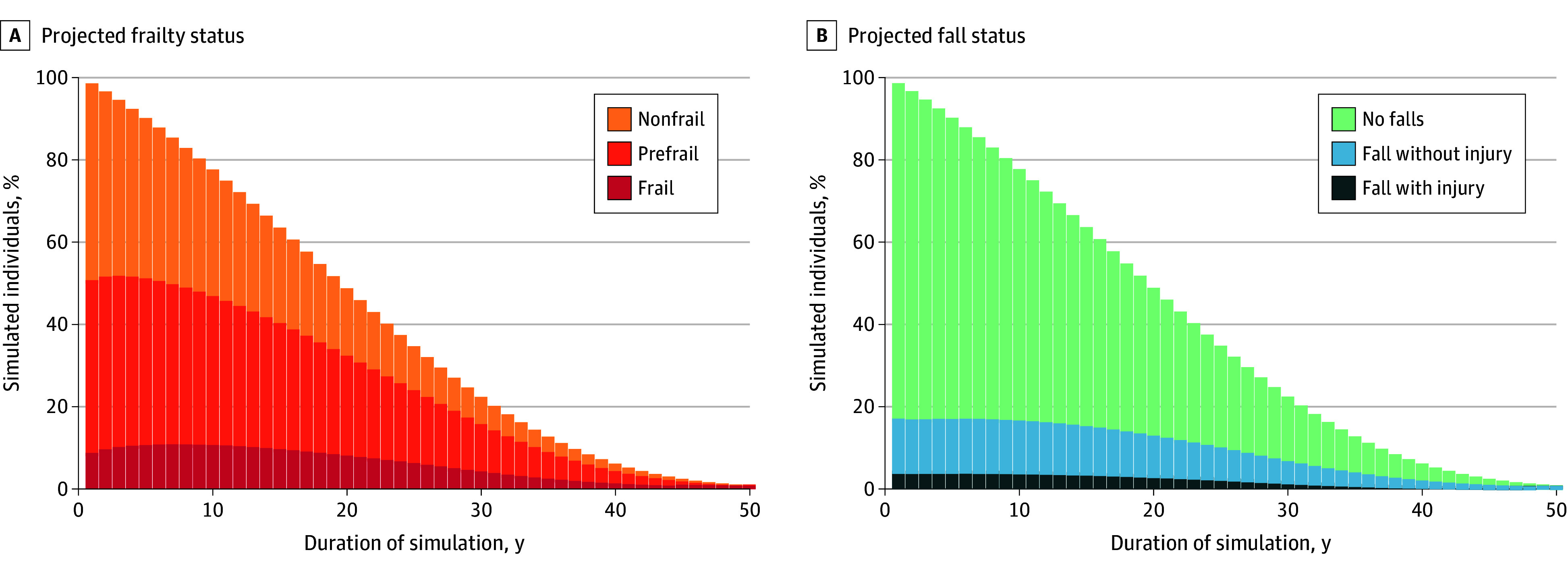
Projected Distribution of Frailty Status and Falls Over Time The figure shows the percentage of simulated individuals in each frailty state (A) and fall state (B) over the duration of the simulation. Individuals started with a mean age of 56 years. Stacked bars do not sum to 100% because of individuals in the simulation who have died.

The model simulated individuals with HIV and viral suppression (mean [SD] age 56 [10] years; 25% female; 41% with prefrailty and 7% with frailty), and results were scaled to reflect the estimated population size of 521 994 PWH. Under the status quo simulation, PWH with viral suppression aged 40 years and over in the United States would have a remaining life expectancy of 20.3 (95% UI, 19.7-20.8) years. They would spend a mean of 9.5 (95% UI, 8.8-10.3) years with prefrailty and 2.4 (95% UI, 1.9-3.1) years with frailty (Figure 2). There would be a mean of 10.1 (95% UI, 8.2-12.1) falls and 1.1 (95% UI, 0.9-1.4) fall-related injuries per person. Remaining quality-adjusted life expectancy (QALE) would be 14.4 (95% UI, 13.9-14.8) QALYs, and there would be $33 000 ($25 300-$43 000) in lifetime per-person frailty and fall-related health care spending. Results by age group are shown in eTable 8 and eFigure 5 in Supplement 1.

### Projections Without Prefrailty, Frailty, and Falls

In simulations without prefrailty, we projected that remaining life expectancy would increase to 20.4 (95% UI, 19.7-20.9) years, QALE would increase to 14.8 (95% UI, 14.3-15.3) QALYs, and per-person frailty and fall-related spending would decrease to $23 400 (95% UI, $16 700-$32 800). In simulations without frailty, remaining life expectancy would increase to 22.9 (95% UI, 20.9-26.6) years, QALE would increase to 16.5 (95% UI, 15.0-19.2) QALYs, and per-person frailty and fall-related spending would decrease to $16 100 (95% UI, $11 400-$22 400). In simulations without falls, remaining life expectancy would increase to 20.7 (95% UI, 20.0-21.2) years, QALE would increase to 14.7 (95% UI, 14.2-15.1) QALYs, and per-person frailty and fall-related spending would decrease to $26 500 (95% UI, $18 900-$36 300) (Table 2).

**Table 2.** Projected Life Expectancy, QALE, and Lifetime Frailty and Fall-Related Costs Among People With HIV and Viral Suppression in the United States^a^

Simulated scenario	Remaining life expectancy (95% UI), y^b^	Remaining QALE (95% UI), y^b^	Lifetime frailty and fall-related costs (95% UI), $
Status quo	20.3 (19.7-20.8)	14.4 (13.9-14.8)	33 000 (25 300-43 000)
No prefrailty	20.4 (19.7-20.9)	14.8 (14.3-15.3)	23 400 (16 700-32 800)
No frailty	22.9 (20.9-26.6)	16.5 (15.0-19.2)	16 100 (11 400-22 400)
No falls	20.7 (20.0-21.2)	14.7 (14.2-15.1)	26 500 (18 900-36 300)

Abbreviations: UI, uncertainty interval; QALE, quality-adjusted life expectancy.

^a^ The status quo simulation represents the projections for people with HIV with the possibility of developing prefrailty, frailty, and falls throughout their lifetimes. The no prefrailty, no frailty, and no falls simulations each remove the respective characteristic to represent a hypothetical scenario without that characteristic.

^b^ Life expectancy and quality-adjusted life expectancy are the remaining life-years or quality-adjusted life-years after the model simulation begins. Individuals in the simulation have a mean age of 56 years at the start.

### Life-Years Lost, QALYs Lost, and Attributable Costs

Compared with the status quo, prefrailty would result in 0.1 (95% UI, 0.0-0.1) life-years lost, 0.4 (95% UI, 0.3-0.6) QALYs lost, and $9600 (95% UI, $6100-$13 800) in spending per person. Frailty would result in 2.6 (95% UI, 0.2-6.4) life-years lost, 2.1 (95% UI, 0.4-4.8) QALYs lost, and $16 900 (95% UI, $9000-$27 200) in spending per person. Falls would result in 0.4 (95% UI, 0.2-0.5) life-years lost, 0.3 (95% UI, 0.2-0.4) QALYs lost, and $6500 (95% UI, $4300-$9200) in spending per person (Table 3).

**Table 3.** Life-Years Lost, QALYs Lost, and Lifetime Costs Attributable to Prefrailty, Frailty, and Falls Among People With HIV and Viral Suppression in the United States

Attributed condition	Life-years lost (95% UI)	QALYs lost (95% UI)	Attributable costs (95% UI), $
**Per person**			
Prefrailty	0.1 (0.0-0.1)	0.4 (0.3-0.6)	9600 (6100-13 800)
Frailty	2.6 (0.2-6.4)	2.1 (0.4-4.8)	16 900 (9000-27 200)
Falls	0.4 (0.2-0.5)	0.3 (0.2-0.4)	6500 (4300-9200)
**Population total**			
Prefrailty	31 000 (16 000-57 000)	214 000 (130 000-292 000)	5.0 (3.2-7.2) billion
Frailty	1 352 000 (84 000-3 336 000)	1 091 000 (209 000-2 500 000)	8.8 (4.7-14.2) billion
Falls	183 000 (120 000-266 000)	141 000 (94 000-198 000)	3.4 (2.2-4.8) billion

Abbreviations: QALYs, quality-adjusted life-years; UI, uncertainty interval.

Scaling these results to the population-level, across the remaining lifetimes of the estimated 521 994 PWH with viral suppression aged 40 years and older in the United States, there would be 214 000 (95% UI, 130 000-292 000) QALYs lost attributable to prefrailty, 1 091 000 (95% UI, 209 000-2 500 000) QALYs lost attributable to frailty, and 141 000 (95% UI, 94 000-198 000) QALYs lost attributable to falls. There would be $5.0 (95% UI, $3.2-$7.2) billion, $8.8 (95% UI, $4.7-$14.2) billion, and $3.4 (95% UI, $2.2-$4.8) billion in spending attributable to prefrailty, frailty, and falls, respectively. eFigure 6 in Supplement 1 shows these results stratified by age group. eTable 9 in Supplement 1 provides outcomes discounted at 3% annually.

### Sensitivity Analyses

In 1-way sensitivity analyses, QALYs lost were most sensitive to the health-related quality-of-life decrements from prefrailty, while lifetime cost estimates were most sensitive to variation in the cost of prefrailty, frailty, and fractures (eTables 10-11 in Supplement 1). Our results were also highly sensitive to the relationship between prefrailty, frailty, and mortality. However, even in the most conservative simulation, in which neither prefrailty nor frailty were directly associated with mortality, across the population there would be 219 000 (95% UI, 136 000-303 000) QALYs lost attributable to prefrailty and 172 000 (95% UI, 125 000-230 000) QALYs lost attributable to frailty (eTable 12 in Supplement 1).

### Scenario Analyses

In simulations based on frailty and fall prevention interventions, we projected that decreasing the risk of prefrailty and frailty by 37% would prevent 444 000 (95% UI, 78 000-1 081 000) QALYs lost and save $4.1 (95% UI, $1.4-$7.0) billion among PWH with viral suppression aged 40 years and older in the United States. Decreasing the risk of falls by 8% was projected to prevent 16 000 (95% UI, 5000-26 000) QALYs lost and to save $365 (95% UI, $104-$679) million (eTable 13 in Supplement 1).

If PWH had prevalences of prefrailty and frailty similar to people without HIV, we projected that PWH with viral suppression aged 40 years and older in the United States would gain 26 000 (95% UI, 5000-52 000) QALYs and would reduce spending by $365 (95% UI, $261-$522) million. If PWH had a fall risk similar to people without HIV, PWH were projected to gain 26 000 (95% UI, 16 000-37 000) QALYs and to reduce spending by $365 (95% UI, $157-$626) million (eTable 14 in Supplement 1).

## Discussion

Our simulation model results suggest that frailty and falls contribute substantially to lower QALE and higher lifetime costs among PWH with viral suppression in the United States. These findings, which combine epidemiologic data on frailty and falls with quality-of-life and cost data, highlight the clinical and economic impact of these important comorbidities. While no policy or intervention will eliminate frailty or falls, our results can be interpreted as an upper bound on preventable health losses and costs from these conditions. The estimates of disease burden highlight the substantial harms associated with frailty and falls and may contribute to making the severity of these conditions salient to patients, clinicians, and policymakers. The estimates may also motivate future work regarding the value of interventions to prevent and treat frailty and falls among PWH.

Compared with studies of people without HIV, our results suggest a higher burden of frailty and falls among PWH. In the Survey of Health, Aging, and Retirement in Europe, a large panel study of the general population in Europe, men aged 55 years were projected to spend approximately 70% of remaining life-years without prefrailty or frailty,^45^ while we projected that PWH (mean age, 56 years; 75% male) would spend 41% of remaining life-years without prefrailty or frailty. Verma and colleagues^46^ estimated that 11% of community-dwelling US adults aged 45 to 64 years fell in the previous 12 months. We projected that among PWH with similar ages, annual fall risk would range from 13% (age 40 years) to 18% (age 60 years).

Frailty and fall prevention programs for PWH could reduce the clinical and economic burden of these conditions. Exercise is directly tied to the frailty phenotype components and is therefore is one of the most effective frailty interventions.^47,48^ In a 12-month randomized study among community-dwelling adults, a physical activity intervention significantly reduced frailty phenotype prevalence to 10% compared with 19% in the control group.^49^ Studies among PWH are more limited, but among PWH enrolled in 24 weeks of moderate- or high-intensity exercise, prefrailty prevalence declined from 75% at baseline (24 of 32 participants) to 44% (12 of 27 participants).^50^ Exercise interventions may also prevent falls. The US Preventive Services Task Force recommends exercise for community-dwelling older adults with an increased risk of falls,^51,52^ and among PWH, a pilot trial^53^ found that a fall prevention program for PWH with alcohol use would be feasible and could reduce falls. Most exercise trials have a duration of 1 year or less,^54,55^ and the duration of benefits from exercise will be an important area of future research. Multifactorial interventions, including medication reviews and deprescription, may also potentially prevent falls and could be investigated among PWH.^56^

In scenario analyses, we compared the status quo with scenarios modeling intervention-based reductions in frailty and falls. We found that intervention-based reductions in frailty incidence and fall risk could result in substantially fewer life-years and QALYs lost. These projections will serve as a foundation for future work that investigates the value of fall and frailty prevention programs for PWH, incorporating intervention costs, shorter-term interventions, and variable adherence rates.

Our results were sensitive to variation in model parameters, particularly the health-related quality-of-life weights associated with prefrailty and the relationship between frailty and mortality. In addition, the uncertainty intervals around the results are wide, reflecting uncertainty in the data that inform our model. For example, we used wide ranges for cost estimates to reflect the fact that these estimates were from studies of people without HIV. Although our results are uncertain, we find a substantial disease burden even in analyses based on conservative assumptions.

### Limitations

Our analysis is subject to several limitations. First, we did not explicitly model osteoporosis, and our analyses therefore implicitly assumed the prevalence of osteoporosis in the cohorts used to estimate model parameters. Second, study participants may differ from PWH who do not enroll in clinical studies, and few participants were older than age 65 years. If study participants experienced lower rates of frailty, falls, and fractures than average or if the frailty state becomes more severe with age, our results likely underestimate overall disease burden. Third, we defined frailty using the modified Fried frailty phenotype used in the ACTG A5322 study. Other frailty metrics, such as the Frailty Index and Veterans Aging Cohort Study Index have been used among PWH and could result in different prevalence estimates.^57,58^ We used the Fried frailty phenotype, as it is a clinically relevant syndrome that is associated with risk of adverse outcomes, combines self-reported and objective assessment, and has been used extensively among PWH.^15,59^ We did not explicitly model the causal paths leading to frailty, as frailty is a syndrome often caused by the combined burden of multiple different stressors. Treatments for frailty, such as exercise, are recommended regardless of the cause.^16,47^

Fourth, we derived costs and injury-related quality-of-life decrements from studies of the general population, as estimates for these parameters were not available among PWH. Our Medicare-based cost estimates may be appropriate, as PWH experience rates of comorbidities at younger ages than people without HIV. In addition, the use of cost estimates from people without HIV is supported by a study of Medicare spending that found that risk-adjusted spending on chronic conditions was similar for beneficiaries without HIV and beneficiaries with HIV receiving antiretroviral therapy.^60^ Fifth, we only modeled costs associated with prefrailty, frailty, and falls and did not include other unrelated health care spending. Sixth, our input parameters are based on associations between frailty and health events such as falls, injuries, and death. However, the relationship between frailty and mortality is complex, and frailty may result from a high comorbidity burden that leads to high mortality risk. We varied the relationship between frailty and mortality in scenario analyses to account for this uncertainty.

## Conclusions

In this decision analytic modeling study, frailty and falls were frequent among PWH and contributed to substantial projected health losses and costs over the next 50 years. Interventions such as exercise programs could improve outcomes, and future work should aim to identify high-value interventions to prevent and treat frailty and falls among PWH.

## Notes

Supplement 1.
eMethods.
**eTable 1.** Sources for Model Input Parameters **eTable 2.** Estimated Number of People With HIV and Viral Suppression in the United States **eTable 3.** Cohort Descriptive Statistics **eTable 4.** Transition Probabilities for Individual Characteristics **eTable 5.** Odds Ratios from Logistic Regression Predicting Fall Risk **eTable 6.** Regression-Estimated Health-Related Quality-of-Life Weights **eTable 7.** Values Varied in 1-Way Sensitivity Analyses **eTable 8.** Life Expectancy by Age Group **eTable 9.** Discounted Life-Years Lost, QALYs Lost, and Lifetime Costs Attributable to Prefrailty, Frailty, and Falls Among People With HIV and Viral Suppression in the United States **eTable 10.** One-Way Sensitivity Analysis for QALYs Lost Attributable to Prefrailty, Frailty, and Falls **eTable 11.** One-Way Sensitivity Analysis for Lifetime Costs Attributable to Prefrailty, Frailty, and Falls **eTable 12.** Sensitivity Analysis With Alternative Prefrailty and Frailty Mortality Hazard Ratios **eTable 13.** Burden of Prefrailty, Frailty, and Falls Among People With HIV and Viral Suppression Relative to Scenarios With Intervention-Based Reductions in Prefrailty, Frailty, and Falls **eTable 14.** Burden of Prefrailty, Frailty, and Falls Among People With HIV and Viral Suppression Relative to People Without HIV **eFigure 1.** Model Validation for Frailty Status **eFigure 2.** Model Validation for Fall-Related Events **eFigure 3.** External Validation of 2-Year Frailty Trajectories **eFigure 4.** External Validation of 2-Year Fall Incidence **eFigure 5.** Projected Frailty and Fall Status by Year of Age **eFigure 6.** Age-Stratified Life-Years Lost, QALYs Lost, and Attributable Costs
eReferences.

Supplement 2.
Data Sharing Statement
