# Supplementary material for: Modeled Health and Economic Burden of Frailty and Falls Among Adults With HIV
Source: JAMA Netw Open. 2026 Jan 21;9(1):e2554809. doi: 10.1001/jamanetworkopen.2025.54809 (PMC12824775; doi:10.1001/jamanetworkopen.2025.54809)
Supplement: Supplement 1. — eMethods. eTable 1. Sources for Model Input Parameters eTable 2. Estimated Number of People With HIV and Viral Suppression in the United States eTable 3. Cohort Descriptive Statistics eTable 4. Transition Probabilities for Individual Characteristics eTable 5. Odds Ratios from Logistic Regression Predicting Fall Risk eTable 6. Regression-Estimated Health-Related Quality-of-Life Weights eTable 7. Values Varied in 1-Way Sensitivity Analyses eTable 8. Life Expectancy by Age Group eTable 9. Discounted Life-Years Lost, QALYs Lost, and Lifetime Costs Attributable to Prefrailty, Frailty, and Falls Among People With HIV and Viral Suppression in the United States eTable 10. One-Way Sensitivity Analysis for QALYs Lost Attributable to Prefrailty, Frailty, and Falls eTable 11. One-Way Sensitivity Analysis for Lifetime Costs Attributable to Prefrailty, Frailty, and Falls eTable 12. Sensitivity Analysis With Alternative Prefrailty and Frailty Mortality Hazard Ratios eTable 13. Burden of Prefrailty, Frailty, and Falls Among People With HIV and Viral Suppression Relative to Scenarios With Intervention-Based Reductions in Prefrailty, Frailty, and Falls eTable 14. Burden of Prefrailty, Frailty, and Falls Among People With HIV and Viral Suppression Relative to People Without HIV eFigure 1. Model Validation for Frailty Status eFigure 2. Model Validation for Fall-Related Events eFigure 3. External Validation of 2-Year Frailty Trajectories eFigure 4. External Validation of 2-Year Fall Incidence eFigure 5. Projected Frailty and Fall Status by Year of Age eFigure 6. Age-Stratified Life-Years Lost, QALYs Lost, and Attributable Costs eReferences. [file jamanetwopen-e2554809-s001.pdf]

Supplemental Online Content

Smith KC, Brown C, Hyle EP, et al. Modeled health and economic burden of frailty and falls among adults with HIV. *JAMA Netw Open.* 2026;9(1):e2554809.
doi:10.1001/jamanetworkopen.2025.54809

eMethods.

eTable 1. Sources for Model Input Parameters

eTable 2. Estimated Number of People With HIV and Viral Suppression in the United States

eTable 3. Cohort Descriptive Statistics

eTable 4. Transition Probabilities for Individual Characteristics

eTable 5. Odds Ratios from Logistic Regression Predicting Fall Risk

eTable 6. Regression-Estimated Health-Related Quality-of-Life Weights

eTable 7. Values Varied in 1-Way Sensitivity Analyses

eTable 8. Life Expectancy by Age Group

eTable 9. Discounted Life-Years Lost, QALYs Lost, and Lifetime Costs Attributable to Prefrailty, Frailty, and Falls Among People With HIV and Viral Suppression in the United States

eTable 10. One-Way Sensitivity Analysis for QALYs Lost Attributable to Prefrailty, Frailty, and Falls

eTable 11. One-Way Sensitivity Analysis for Lifetime Costs Attributable to Prefrailty, Frailty, and Falls

eTable 12. Sensitivity Analysis With Alternative Prefrailty and Frailty Mortality Hazard Ratios

eTable 13. Burden of Prefrailty, Frailty, and Falls Among People With HIV and Viral Suppression Relative to Scenarios With Intervention-Based Reductions in Prefrailty, Frailty, and Falls

eTable 14. Burden of Prefrailty, Frailty, and Falls Among People With HIV and Viral Suppression Relative to People Without HIV

eFigure 1. Model Validation for Frailty Status

eFigure 2. Model Validation for Fall-Related Events

eFigure 3. External Validation of 2-Year Frailty Trajectories

eFigure 4. External Validation of 2-Year Fall Incidence

eFigure 5. Projected Frailty and Fall Status by Year of Age

eFigure 6. Age-Stratified Life-Years Lost, QALYs Lost, and Attributable Costs

eReferences.

This supplemental material has been provided by the authors to give readers additional information about their work.

© 2026 Smith KC et al. *JAMA Network Open*.

eMethods

Model Structure

The Frailty Policy Model is a simulation model of frailty and falls among people with HIV (PWH) in the United States. Each year in the simulation, simulated individuals transition between health states, which include non-fracture fall injuries, fall-related fractures, and death. Individual characteristics, such as frailty, are tracked at the person-level and determine the annual risk of one or multiple falls. We simulated 2,000,000 individuals in each analysis and calculated per-person outcomes or scaled outcomes to the number of PWH with viral suppression in the United States aged 40 years and older in 2022. The analysis used version 1.0 of the Frailty Policy Model, which was coded in R (version 4.4.2). This version of the model was developed using literature on microsimulation modeling in R from the Decision Analysis in R for Technologies in Health (DARTH) group.[1,2]

Data Sources

The model parameters are primarily estimated from two longitudinal studies of PWH: (1) the Advancing Clinical Therapeutics Globally for HIV/AIDS and Other Infections (ACTG) A5322 Study: Long-Term Follow-up of Older HIV-infected Adults in the ACTG: Addressing Issues of Aging, HIV Infection and Inflammation,[3] and (2) the Multicenter AIDS Cohort Study (MACS) / Women's Interagency HIV Study (WIHS) Combined Cohort Study (MWCCS). The ACTG A5322 Study is a prospective observational study of 1,035 aging adults with HIV aged 40 years and older. The MWCCS integrates two longitudinal studies of men and women with HIV, and we used both historic MACS/WIHS data from the separate studies and the combined study data.[4] eTable 1 provides an overview of the model parameters estimated from each study.

Model Outcomes

The outcomes for this analysis are life-years lost, quality-adjusted life-years (QALYs) lost, and lifetime frailty and fall-related costs using a healthcare sector perspective. We report undiscounted outcomes in the base case analyses because our results are a description of disease burden rather than an economic evaluation to guide resource allocation. We present outcomes discounted at 3% annually in sensitivity analyses.

Model Cohort

We projected results for PWH with viral suppression in the United States aged 40 years and older, and our population-level results are scaled to reflect the number of PWH with viral suppression in the United States aged 40 years and older in 2022. We calculated the size of this cohort using published data from the CDC reporting the number of PWH by age and sex at birth and viral suppression status.[5,6] eTable 2 shows the final population estimates used in the analyses.

Risk Factor Distributions

We assigned fall and fracture risk factors to simulated individuals at model initialization based on the distribution of risk factors in the ACTG A5322 study. We used data from 961 participants who reported values for all relevant baseline variables and fall history at the 1-year visit. As the simulation model cohort is based on PWH with viral suppression, we excluded participants with a viral load over 200 copies/mL at over 50% of study visits. In uncertainty analyses, we sampled

© 2026 Smith KC et al. *JAMA Network Open*.

these parameters from 1,000 bootstrapped datasets to preserve the correlation across the parameters. eTable 3 shows the characteristics of the starting cohort.

Transition Probabilities

Individual-Level Characteristics

eTable 4 describes the probabilities used in the simulation model to update individual-level characteristics each year. These transition probabilities were derived from the ACTG A5322 study, excluding participants with a viral load over 200 copies/mL at over 50% of study visits. In uncertainty analyses, probabilities were drawn from beta distributions. We determined the parameters for beta distributions by estimating probabilities with a logistic regression with generalized estimating equation (GEE) adjustments. We then used method of moments calculations with the regression-estimated mean and standard error. This accounts for the within-person correlation during the longitudinal study.

We assumed that once an individual was diagnosed with depression or chronic pain, they had the diagnosis for the remainder of their lifetime. We assumed that transitions between frailty statuses occurred at the midpoint when two different frailty statuses were reported. For uncertainty estimates around frailty transitions, we used Poisson distributions and converted rates to probabilities.

Our estimated frailty transitions are consistent with those in published literature. For example, the SEPTAVIH study of PWH in France reported one-year frailty transitions. The probability of progression from non-frailty to pre-frailty was 43%, and the probability of progression from pre-frailty to frailty was 11%. The probability of improvement from pre-frailty to non-frailty was 14%, and the probability of improvement from frailty to pre-frailty was 47%.[7] These are similar to our estimates (25%, 15%, 13%, and 39%, respectively), and the comparison suggests that we may underestimate the development of pre-frailty, making our results a more conservative estimate of disease burden. SEPTAVIH participants were aged 70 or older (median 73), which supports the use of our estimated frailty transitions to project beyond age 65 years.

Falls, Injuries, and Fractures

We developed a logistic regression model to predict an individual's probability of experiencing falls using data from the ACTG A5322 study (eTable 5). Falls were characterized using the definition used in both MWCCS and ACTG A5322: an unexpected event in which an individual loses balance and lands on the floor, ground, or a lower level, or hits an object. Participants in A5322 reported a 12-month history of falls biannually, and our outcome variable was whether a participant reported any falls in the past year. To account for correlation within individuals, we used a logistic regression with GEE adjustment. We included dependent variables that had previously been identified as fall risk factors, including age, sex, opioids and psychotropics, comorbidities, smoking status, and history of falls in the previous year.

The probability of a non-fracture injury or a fracture following a fall (Table 1) was also estimated from self-reported fall injuries in the ACTG A5322 study using logistic regression. In probabilistic sensitivity analyses, we drew values from a multivariate normal distribution using the regression coefficients and the variance-covariance matrix.

Each year of the simulation, individuals faced age- and sex-stratified probabilities of mortality. These were based on estimates from the Cost-Effectiveness of Preventing AIDS Complications (CEPAC) model, and the probabilities represent mortality among PWH taking antiretroviral therapy, accounting for the increased risk of mortality among PWH due to non-HIV-related

© 2026 Smith KC et al. *JAMA Network Open*.

factors.[8] Frailty increased the risk of mortality by 3.19 (95% CI 1.02, 9.98) based on a prospective study of frailty and mortality among PWH.[9] Hip fractures increased mortality in the six months after the fracture (hazard ratio of 6.28, (95% CI: 4.82, 8.19)).[10] We implemented this six-month hazard ratio in the annual model cycle by assuming a constant mortality rate throughout the year and applying the hazard ratio to the rate for half the year. We modeled unexplained variance in mortality through a probabilistic approach as error is incorporated through each simulated person facing a probability of mortality in each year (i.e., if there are two simulated people with identical characteristics and identical probabilities of mortality, one may die in a given year and another may not).

Costs

All costs were adjusted to 2023 USD using the Personal Health Care (PHC) index.[11] The costs of pre-frailty and frailty were from a 2023 study of the incremental health care costs predicted by frailty status.[12] Ensrud and colleagues estimated healthcare costs for 8,165 Medicare fee-for-service beneficiaries enrolled in the Study of Osteoporotic Fractures, the Osteoporotic Fractures in Men Study, the Health, Aging and Body Composition Study, and the National Health and Aging Trends Study. They reported the additional costs predicted by phenotypic frailty, adjusting for CMS HCC score, multimorbidity (a count of 27 chronic conditions), and a claims-based frailty index. These costs include healthcare costs paid by Medicare (hospital, skilled-nursing and inpatient rehabilitation facility stays, outpatient care, medical equipment, and home healthcare). For men, pre-frailty did not statistically significantly increase costs, so we assumed pre-frailty would have no incremental costs among men.

Fracture costs were from an analysis of Medicare spending on osteoporotic fractures in 2016.[13] Hansen and colleagues reported the estimated incremental cost for ten fracture types by comparing Medicare beneficiaries with the given fracture to matched controls. We used their estimated costs of $45,389 and $18,520 for hip and spine fractures, respectively. For the wrist/arm fracture category and the other fracture category, we used averages weighted by the distribution of fall-related fractures in the MWCCS. The wrist/arm category was a weighted average of distal radius/ulna fractures (51%, $9,700), ulna/radius fractures (28%, $10,400), and humerus fractures (21%, $17,200). Other fractures included foot (23%, $9,700), ankle/leg (30%, $16,700), rib (20%, $13,500), knee (11%, $9,700), hand (6%, $9,700), collarbone (3%, $12,000), pelvis (1%, $23,800), elbow (2%, $9,700), scapula (2%, $12,000), and other lower limb (1%, $16,700). The costs listed above assumed that foot, knee, hand, and elbow fractures had the same cost as wrist fractures, scapula had the same cost as clavicle fractures, and that other lower limb fractures had the same cost as leg fractures, as costs were not available for these specific fracture types. The costs listed above are the original costs in 2016 USD, and we adjusted to 2023 USD.

The cost of non-fracture injuries was calculated as a weighted average of the cost of fall-related injuries treated in emergency departments and the cost of urgent care visits. Based on a study of older adults who received medical attention for fall injuries,[14] we estimated that 44% of fall-related injuries would be treated in the emergency department, while the remaining 56% would be treated in an urgent care setting. The cost of injuries treated in an emergency department was calculated using a study of the average medical costs of emergency department-treated injuries in the United States using MarketScan claims data.[15] We weighted the cost of different injury types using distributions of non-fatal fall injuries treated in emergency departments.[16] The cost of an urgent care visit was $200 (2023 USD), based on the cost of a low-acuity urgent care center visit in national managed care plan.[17]

© 2026 Smith KC et al. *JAMA Network Open*.

The cost estimates described above are from people without HIV. In a study of Medicare spending, risk-adjusted spending on non-HIV chronic conditions was similar for beneficiaries without HIV and beneficiaries with HIV receiving antiretroviral therapy,[18] which suggests that spending on frailty, injuries, and fractures may be similar among PWH with viral suppression and people without HIV. Nevertheless, there is still uncertainty around whether our cost estimates reflect costs for PWH. We incorporated this uncertainty by modeling a wide distribution around costs using a gamma distribution with a mean equal to the estimates described above and a standard deviation equal to 20% of the mean.

Health-Related Quality-of-Life Weights

Each health state in the simulation is associated with a health-related quality-of-life weight which ranges from 0 (death) to 1 (perfect health). QALYs are calculated by multiplying the health-related quality-of-life weight for a state by the years spent in that state.

We estimated health-related quality-of-life weights for health states using responses to the Medical Outcomes Study 36-Item Short Form Survey (SF-36) from MWCCS participants.[19] We used six scales scored from 0 to 100 measuring physical function, role function, social function, pain, emotional wellbeing, and health perception. To map these values to a health-related quality-of-life weight ranging from 0 to 1, we categorized responses by quantiles and used an algorithm proposed by Brazier and colleagues.[20] We then used a linear regression to model health-related quality-of-life weights as a function of age, sex, frailty status, number of comorbidities, depression, and chronic pain. We tested the addition of interaction terms between frailty status and the other variables, and the final model included an interaction between frailty status and chronic pain, as well as frailty status and sex (eTable 6). We also tested models with controls for insurance status (Medicare, Medicaid, private, other). As the inclusion of insurance status did not change the values of the other coefficients, we did not include insurance status in the final model. In probabilistic sensitivity analyses, we drew values from a multivariate normal distribution using the point estimates in eTable 6 and the variance-covariance matrix.

We derived health-related quality-of-life decrements from injuries and fractures using a study of QALY losses due to injury in the United States,[21] as these events were too infrequent to assess with the MWCCS data.

Sensitivity and Scenario Analyses

eTable 7 describes the parameters varied in one-way deterministic sensitivity analyses. We also conducted a sensitivity analysis varying the relationship between pre-frailty/frailty and mortality using alternative hazard ratios from published literature. We used estimates from a study of PWH in the Veterans Aging Cohort Study that reported hazard ratios of 1.44 and 1.75 for pre-frailty and frailty, respectively.[22] We also used estimates from a published analysis of PWH in the ACTG A5322 study, which found no significant association between baseline pre-frailty or baseline frailty and subsequent mortality.[23] We therefore assumed hazard ratios of 1 for these analyses.

In addition, we modeled scenarios based on two alternative comparisons. The first set of simulations compared the status quo to simulations with frailty and fall risk reductions based on intervention efficacy. In these scenarios, the risk of pre-frailty or frailty was reduced by 37% based on a meta-analysis of exercise interventions to prevent physical frailty, which reported a relative risk of 0.63 (95% CI 0.47–0.84).[24] Fall risk was reduced by 8% based on the United States Preventive Services Task Force review of exercise interventions for fall prevention, which

© 2026 Smith KC et al. *JAMA Network Open*.

reported a relative risk of 0.92 (95% CI, 0.87-0.98).[25] We assumed these risk reductions would occur throughout an individual's lifetime.

The second set of simulations compared the status quo to simulations with the prevalence of pre-frailty and frailty and fall risk set to that of people without HIV. We based estimates for people without HIV on nationally representative studies in the United States and large population-based cohort studies, adjusted to reflect the age distribution of PWH in the United States.[26-29] For adults ages <50, 50-64, and ≥65 years, we estimated pre-frailty prevalences of 37%, 37%, and 43%, respectively, and frailty prevalences of 3%, 3%, and 10%, respectively. For adults ages <55, 55-64, and ≥65 years, we estimated that the annual risk of falls would be 10%, 12%, and 26%, respectively.

Model Validation

In internal model validation analyses, we simulated cohorts based on the ACTG A5322 study. We compared years spent with pre-frailty and frailty, and rates of falls, injuries, and fractures to outcomes observed in the longitudinal study. The study data were from participants who had complete information for covariates of interest at baseline and, for the falls, injuries, and fractures validations, completed the fall questionnaire at the 1-year visit. We compared values at baseline through the year 5 follow-up visit. We ran 1000 simulations using values drawn from the distributions described in Table 1, and we report the results with the mean of the 1000 simulations and the 95% uncertainty interval.

All validation outcomes were within the observed 95% confidence intervals (eFigures 1 – 2). In ACTG A5322, 42% (95% CI 39%, 44%) of years were spent with pre-frailty, 7% (95% CI 6% - 8%) of years were spent with frailty, and there were 37 (95% CI 32, 42) falls per 100 person-years. In the simulations of ACTG A5322, we projected that 42% (95% UI 40%, 43%) of years would be spent with pre-frailty, 8% (95% UI 7%, 9%) of years would be spent with frailty, and there would be 33 (95% UI 29 - 37) falls per 100 person-years.

In external validation analyses, we compared model projections to independent data sources. We externally validated pre-frailty and frailty against frailty transitions reported in the AGE$_h$IV study.[30] We externally validated fall incidence against falls reported in the MACS Bone Strength Substudy and falls reported in WIHS over two years.[31,32] The MACS Bone Strength Substudy and WIHS comparisons are external, as we did not use MWCCS data to parameterize any fall-related model parameters. When the external sources did not report 95% confidence intervals for an outcome, we calculated confidence intervals based on the reported number of observations with/without the event, and we compared the simulated results to the observed point estimate and 95% confidence interval. We report the results of these external validations in eFigures 3 and 4. The model slightly underestimated the incidence of falls relative to the observed data, which suggests that our results may be conservative estimates of fall burden.

Supplemental Results

eTable 8 and eFigures 5-6 show the base case results stratified by age. eTable 9 reports the results of a sensitivity analysis discounting the outcomes at 3% annually. eTables 10-11 show the results of deterministic one-way sensitivity analyses, and eTable 12 shows the results of analyses varying the hazard ratio for mortality associated with pre-frailty and frailty. eTables 13 and 14 show the results of scenario analyses, comparing the status quo to intervention-based reductions in frailty and falls (eTable 13) and prevalence of frailty and falls among people without HIV (eTable 14).

© 2026 Smith KC et al. *JAMA Network Open*.

eTable 1. Sources for Model Input Parameters

Study	Parameters
ACTG A5322	Cohort characteristics at baseline<br>Annual probability of changing frailty status<br>Annual probability of changing smoking status<br>Annual probability of opioid or psychotropic prescription<br>Annual probability of developing a new comorbidity<br>Annual probability of falls<br>Probability of injury or fracture conditional on a fall
MACS/WIHS Combined Cohort Study	Fracture sites (hip, spine, wrist/arm, other)<br>Health-related quality-of-life weights
Published Literature	Mortality increases for frailty state[9]<br>Mortality increases for hip fracture state[10]<br>Costs[12,13,15]<br>Injury and fracture quality-of-life decrements[21]<br>Efficacy of frailty and fall prevention interventions[24,25]<br>Pre-frailty, frailty, and falls among people without HIV[26-29]

The table shows the sources for model parameters. Additional details are included in Table 1 and the methods section of the main text and the eMethods section of the Supplement.

© 2026 Smith KC et al. *JAMA Network Open*.

eTable 2. Estimated Number of People With HIV and Viral Suppression in the United States

Age in 2022	Male	Female	Total
40-44	50,037	16,847	66,884
45-49	47,964	19,471	67,435
50-54	63,363	22,870	86,233
55-59	82,461	25,108	107,569
60-64	70,319	21,520	91,839
65-69	41,834	12,961	54,795
70+	36,222	11,017	47,239
Total	392,200	129,794	521,994

The number of people with HIV and viral suppression aged 40 years and older was estimated from the CDC 2022 HIV Surveillance Report.[5,6]

© 2026 Smith KC et al. *JAMA Network Open*.

eTable 3. Cohort Descriptive Statistics

Characteristic	% Simulated Individuals (95% UI)
Smoking Status	
Non-smoker	41% (38%, 45%)
Prior smoker	34% (31%, 37%)
Current Smoker	25% (22%, 27%)
Comorbidity Status	
0 Comorbidities	64% (60%, 69%)
1 Comorbidity	27% (24%, 30%)
2+ Comorbidities	9% (6%, 11%)
Depression	31% (28%, 34%)
Chronic Pain	17% (15%, 20%)
Frailty Status	
Non-Frail	51% (48%, 55%)
Pre-Frail	41% (38%, 45%)
Frail	7% (6%, 9%)
Fall-Risk Increasing Drugs Prescribed	
None	62% (59%, 65%)
Opioids Only	2% (1%, 3%)
Psychotropic Only	32% (29%, 35%)
Opioid and Psychotropic	4% (3%, 5%)

Abbreviations: UI, uncertainty interval.

The table shows characteristics of the modeled cohort at model initialization. The point estimate shown is the mean and the 95% UI is from 1000 bootstrapped samples of the ACTG A5322 study. Percentages may not sum to 100% due to rounding.

© 2026 Smith KC et al. *JAMA Network Open*.

eTable 4. Transition Probabilities for Individual Characteristics

Probability	Point Estimate	Distribution
P(New Comorbidity)	6%	Beta (211, 3504)
P(New Chronic Pain | 0 comorbidities)	2%	Beta (52, 2075)
P(New Chronic Pain | 1 comorbidity)	3%	Beta (25, 896)
P(New Chronic Pain | 2+ comorbidities)	6%	Beta (18, 284)
P(Depression | No Chronic Pain)	2%	Beta (59, 2278)
P(Depression | Chronic Pain)	4%	Beta (18, 400)
P(Prescribed Opioid)		
Not currently prescribed an opioid, no new pain	1%	Beta (38, 3803)
Not currently prescribed an opioid, new pain	43%	Beta (42, 57)
Currently prescribed an opioid	80%	Beta (241, 66)
P(Prescribed Psychotropic)		
Not currently prescribed a psychotropic, no new depression	3%	Beta (72, 2510)
Not currently prescribed a psychotropic, new depression	76%	Beta (21, 7)
Currently prescribed a psychotropic, no new depression	93%	Beta (1259, 104)
Currently prescribed a psychotropic, new depression	85%	Beta (42, 8)

Probabilities and distributions were estimated from the ACTG A5322 study, excluding participants with a viral load above 200 at over half of study visits.

© 2026 Smith KC et al. *JAMA Network Open*.

eTable 5. Odds Ratios from Logistic Regression Predicting Fall Risk

**Characteristic**	**Odds Ratio (95% CI)**
Age	
55 – 64	1.10 (0.89, 1.37)
65+	1.59 (1.15, 2.20)
Female	1.32 (1.04, 1.68)
Frailty Status	
Pre-Frail	1.70 (1.37, 2.11)
Frail	2.75 (1.93, 3.90)
Fall-Risk Increasing Drugs	
Psychotropic	1.19 (0.96, 1.49)
Psychotropic and Opioid	1.79 (1.21, 2.64)
Comorbidities	
1 Comorbidity	1.16 (0.93, 1.45)
2+ Comorbidities	1.38 (1.01, 1.87)
Smoking Status	
Prior Smoker	1.25 (0.99, 1.58)
Current Smoker	1.66 (1.29, 2.14)
Fall History	
1-2 Falls	3.51 (2.76, 4.45)
3+ Falls	15.9 (10.8, 23.3)

Abbreviations: CI, confidence interval.

Values are odds ratios from a logistic regression with generalized estimating equation adjustment that used data from the ACTG A5322 study, excluding participants with a viral load above 200 at over half of study visits.

© 2026 Smith KC et al. *JAMA Network Open*.

eTable 6. Regression-Estimated Health-Related Quality-of-Life Weights

Characteristic	Coefficient (95% CI)
Constant	0.74 (0.71, 0.76)
Age	0.001 (0.000, 0.001)
Female	-0.02 (-0.01, 0.010)
Depression	-0.05 (-0.06, -0.04)
Chronic Pain	-0.004 (-0.02, 0.02)
Frailty Status	
Pre-Frailty	-0.02 (-0.03, -0.01)
Frailty	-0.06 (-0.08, -0.04)
Number of Comorbidities	
1 Comorbidity	-0.01 (-0.02, -0.002)
2+ Comorbidities	-0.01 (-0.02, -0.01)
Interaction Terms	
Pre-Frailty x Female	-0.02 (-0.04, -0.01)
Frailty x Female	-0.04 (-0.06, -0.01)
Pre-Frailty x Chronic Pain	-0.03 (-0.05, -0.01)
Frailty x Chronic Pain	-0.06 (-0.09, -0.04)

Abbreviations: CI, confidence interval.

Values were estimated from a linear regression using data from the MACS/WIHS Combined Cohort Study. Pre-frailty and frailty cut points were defined using the MACS/WIHS Combined Cohort Study derived cut points.

© 2026 Smith KC et al. *JAMA Network Open*.

eTable 7. Values Varied in 1-Way Sensitivity Analyses

1-Way Sensitivity Analysis	Parameters Varied	Low Estimate	High Estimate	Source
Non-Frail to Pre-Frail Transition Probability	Age <50	20%	34%	±25% of base case estimate
	Age 50-64	16%	28%	
	Age 65+	19%	31%	
Pre-Frail to Frail Transition Probability	Age <50	6%	9%	±25% of base case estimate
	Age 50-64	7%	12%	
	Age 65+	10%	16%	
Pre-Frail to Non-Frail Transition Probability	Age <50	25%	41%	±25% of base case estimate
	Age 50-64	17%	29%	
	Age 65+	10%	16%	
Frail to Pre-Frail Transition Probability	Age <50	33%	55%	±25% of base case estimate
	Age 50-64	29%	49%	
	Age 65+	29%	49%	
Probability of Falls	Probability of falls for someone with no fall risk factors	3.9%	6.1%	95% CI of intercept in logistic regression. The effects of fall risk factors (shown in eTable 5) were held at base case values.
Hip Fracture Mortality	Hazard ratio	4.82	8.19	95% confidence interval from Tosteson et al.[10]
Pre-Frailty and Frailty Quality-of-Life Decrements	Pre-frailty, male, no chronic pain	0.01	0.03	Estimated from our analysis of MWCCS data (95% CIs reported in eTable 6).
	Pre-frailty, male, with chronic pain	0.01	0.08	
	Pre-frailty, female, No chronic pain	0.01	0.07	
	Pre-frailty, female, with chronic pain	0.02	0.12	
	Frailty, male, no chronic pain	0.04	0.08	
	Frailty, male, with chronic pain	0.08	0.16	
	Frailty, female, no chronic pain	0.05	0.14	
	Frailty, female, with chronic pain	0.09	0.22	
Injury Quality-of-Life Decrement	Non-fracture injury, Year 1 / year 2	0.01 / 0	0.02 / 0.01	Estimated from 95% CIs in published literature.[21] Low estimate assumed no effect in year 2.

© 2026 Smith KC et al. *JAMA Network Open*.

1-Way Sensitivity Analysis	Parameters Varied	Low Estimate	High Estimate	Source
Fracture Quality-of-Life Decrement	Hip fracture, Year 1 / year 2	0.04 / 0	0.09 / 0.03	Estimated from 95% CIs in published literature.[21] Low estimate assumed no effect in year 2.
	Spine fracture, Year 1 / year 2	0.03 / 0	0.07 / 0.08	
	Wrist/arm fracture, Year 1 / year 2	0.01 / 0	0.05 / 0.04	
	Other fracture, Year 1 / year 2	0.03 / 0	0.05 / 0.03	
Pre-Frailty and Frailty Annual Cost	Pre-frailty cost, male	$0	$0	Estimated from 95% CIs in published literature.[12]
	Frailty cost, male	$3,400	$9,900	
	Pre-frailty cost, female	$1,800	$5,100	
	Frailty cost, female	$6,800	$11,500	
Injury Cost	Non-fracture injury	$2,000	$4,600	±40% of base case cost estimate
Fracture Costs	Hip fracture	$29,900	$69,700	±40% of base case cost estimate
	Spine fracture	$12,200	$28,400	
	Wrist/arm fracture	$7,600	$17,600	
	Other fracture	$8,600	$20,000	

Abbreviations: CI, confidence interval. MWCCS, MACS/WIHS Combined Cohort Study.

The table shows the parameters varied in each of the 1-way sensitivity analysis. The low and high values of parameters are based on 95% confidence intervals, with the exceptions of frailty transition probabilities and injury and fracture costs. The frailty transition probabilities were varied ±25% from the base case estimate and injury and fracture cost estimates were varied ±40% of the base case estimate because the original confidence intervals were narrow, and we aimed to assess additional uncertainty in the parameter (for example, because costs estimates were from studies of people without HIV).

© 2026 Smith KC et al. *JAMA Network Open*.

eTable 8. Life Expectancy by Age Group

**Age Group**	**Life Expectancy, Years (95% UI)**
40 − 44	30.7 (30.0, 31.4)
45 − 49	27.0 (26.3, 27.6)
50 − 54	23.3 (22.6, 23.9)
55 − 59	19.8 (19.2, 20.4)
60 − 64	16.5 (15.9, 17.1)
65 − 69	13.5 (12.8, 14.0)
70+	9.7 (9.3, 10.3)

Abbreviations: UI, uncertainty interval.

The table shows the remaining life expectancy for people with HIV with viral suppression by age group. The average age of each group is the midpoint value.

© 2026 Smith KC et al. *JAMA Network Open*.

eTable 9. Discounted Life-Years Lost, QALYs Lost, and Lifetime Costs Attributable to Prefrailty, Frailty, and Falls Among People With HIV and Viral Suppression in the United States

	Per Person (95% UI)		
**Attributable to**	**Life-Years Lost**	**QALYs Lost**	**Attributable Costs**
Pre-Frailty	0.03<br>(0.01 – 0.06)	0.26<br>(0.16 – 0.36)	$6,600<br>($4,200 – $9,500)
Frailty	1.29<br>(0.08 – 3.13)	1.08<br>(0.24 – 2.39)	$11,500<br>($6,800 – $18,000)
Falls	0.18<br>(0.12 – 0.27)	0.15<br>(0.10 – 0.21)	$4,400<br>($2,900 – $6,100)
	**Population Total (95% UI)**		
**Attributable to**	**Life-Years Lost**	**QALYs Lost**	**Attributable Costs**
Pre-Frailty	16,000<br>(5,000 – 31,000)	136,000<br>(84,000 – 188,000)	$3.4B<br>($2.2B – $5.0B)
Frailty	673,000<br>(42,000 – 1,634,000)	564,000<br>(125,000 – 1,248,000)	$6.0B<br>($3.5B – $9.4B)
Falls	94,000<br>(63,000 – 141,000)	78,000<br>(52,000 – 110,000)	$2.3B<br>($1.5B – $3.2B)

Abbreviations: UI, uncertainty interval; QALYs, quality-adjusted life-years; B, billions.

The table shows discounted (3% annually) life-years lost, QALYs lost, and lifetime costs attributable to pre-frailty, frailty, and falls among people with HIV with viral suppression aged 40 and over in the United States. The top section of the table shows per-person values, and the bottom section shows the population total scaling the per-person results to the estimated population size (521,994 people).

© 2026 Smith KC et al. *JAMA Network Open*.

eTable 10. One-Way Sensitivity Analysis for QALYs Lost Attributable to Prefrailty, Frailty, and Falls

Percentage Change in QALYs Lost Compared to the Base Case			
**Parameter**	**QALYs Lost Attributable to**	**Parameter set to low estimate**	**Parameter set to high estimate**
Non-Frail to Pre-Frail Transition Probability	Pre-Frailty	−10%	7%
	Frailty	−9%	7%
	Falls	<5%	<5%
Pre-Frail to Frail Transition Probability	Pre-Frailty	<5%	−8%
	Frailty	−20%	15%
	Falls	<5%	<5%
Pre-Frail to Non-Frail Transition Probability	Pre-Frailty	5%	−10%
	Frailty	5%	−7%
	Falls	<5%	<5%
Frail to Pre-Frail Transition Probability	Pre-Frailty	−5%	<5%
	Frailty	16%	−12%
	Falls	<5%	<5%
Probability of Falls	Pre-Frailty	<5%	<5%
	Frailty	<5%	<5%
	Falls	−18%	20%
Hip Fracture Mortality Hazard Ratio	Pre-Frailty	<5%	<5%
	Frailty	<5%	<5%
	Falls	−20%	25%
Pre-Frailty and Frailty Quality-of-Life Decrement	Pre-Frailty	−60%	59%
	Frailty	<5%	<5%
	Falls	<5%	<5%
Injury Quality-of-Life Decrement	Pre-Frailty	<5%	<5%
	Frailty	<5%	<5%
	Falls	<5%	<5%
Fracture Quality-of-Life Decrement	Pre-Frailty	<5%	<5%
	Frailty	<5%	<5%
	Falls	<5%	<5%

<−50%
−50 to −25%
−25 to −10%
−10 to 10%
10 to 25%
25 to 50%
>50%

Abbreviations: QALY, quality-adjusted life-year.

The table shows the percentage change in QALYs lost attributable to either pre-frailty, frailty, or falls varying each model parameter. Values used in the sensitivity analyses are shown in eTable 7.

© 2026 Smith KC et al. *JAMA Network Open*.

eTable 11. One-Way Sensitivity Analysis for Lifetime Costs Attributable to Prefrailty, Frailty, and Falls

Percentage Change in Lifetime Costs Compared to the Base Case			
**Parameter**	**Costs Attributable to**	**Parameter set to low estimate**	**Parameter set to high estimate**
Non-Frail to Pre-Frail Transition Probability	Pre-Frailty	−10%	7%
	Frailty	−9%	6%
	Falls	<5%	<5%
Pre-Frail to Frail Transition Probability	Pre-Frailty	5%	−7%
	Frailty	−20%	16%
	Falls	<5%	<5%
Pre-Frail to Non-Frail Transition Probability	Pre-Frailty	6%	−8%
	Frailty	5%	−7%
	Falls	<5%	<5%
Frail to Pre-Frail Transition Probability	Pre-Frailty	-5%	<5%
	Frailty	17%	−13%
	Falls	<5%	<5%
Probability of Falls	Pre-Frailty	<5%	<5%
	Frailty	<5%	<5%
	Falls	−19%	22%
Hip Fracture Mortality Hazard Ratio	Pre-Frailty	<5%	<5%
	Frailty	<5%	<5%
	Falls	<5%	−5%
Pre-Frailty and Frailty Annual Costs	Pre-Frailty	−39%	39%
	Frailty	−41%	41%
	Falls	5%	−5%
Injury Costs	Pre-Frailty	<5%	<5%
	Frailty	<5%	<5%
	Falls	−16%	16%
Fracture Costs	Pre-Frailty	<5%	<5%
	Frailty	<5%	<5%
	Falls	−28%	28%

Color key:

- <−50%
- −50 to −25%
- −25 to −10%
- −10 to 10%
- 10 to 25%
- 25 to 50%
- >50%

The table shows the percentage change in lifetime costs attributable to either pre-frailty, frailty, or falls varying each model parameter. Values used in the sensitivity analyses are shown in eTable 7.

© 2026 Smith KC et al. *JAMA Network Open*.

eTable 12. Sensitivity Analysis With Alternative Prefrailty and Frailty Mortality Hazard Ratios

Per Person (95% UI)				
**Mortality Hazard Ratio**	**Attributable to**	**Life-Years Lost**	**QALYs Lost**	**Attributable Costs**
Pre-Frailty: 1.44 Frailty: 1.75	Pre-Frailty	1.59 (0.99 – 2.30)	1.50 (1.01 – 2.02)	$7,200 ($3,400 – $11,700)
	Frailty	0.84 (0.35 – 1.44)	0.87 (0.50 – 1.31)	$20,200 ($13,400 – $29,000)
No increased mortality (hazard ratio = 1)	Pre-Frailty	0.08 (0.04 – 0.12)	0.42 (0.26 – 0.58)	$9,700 ($6,200 – $13,900)
	Frailty	0.06 (0.03 – 0.10)	0.33 (0.24 – 0.44)	$22,600 ($15,100 – $32,400)

Population Total (95% UI)				
**Mortality Hazard Ratio**	**Attributable to**	**Life-Years Lost**	**QALYs Lost**	**Attributable Costs**
Pre-Frailty: 1.44 Frailty: 1.75	Pre-Frailty	830,000 (517,000 – 1,201,000)	783,000 (527,000 – 1,054,000)	$3.8B ($1.8B – $6.1B)
	Frailty	438,000 (183,000 – 752,000)	454,000 (261,000 – 684,000)	$10.5B ($7.0B – $15.1B)
No increased mortality (hazard ratio = 1)	Pre-Frailty	42,000 (21,000 – 63,000)	219,000 (136,000 – 303,000)	$5.1B ($3.2B – $7.3B)
	Frailty	31,000 (16,000 – 52,000)	172,000 (125,000 – 230,000)	$11.8B ($7.9B – $16.9B)

Abbreviations: UI, uncertainty interval; QALYs, quality-adjusted life-years; B, billions.

The table shows results from scenario analyses using alternative estimates for the relationship between pre-frailty and mortality and frailty and mortality. Akgun et al. reported hazard ratios of 1.44 and 1.75 for pre-frailty and frailty, respectively.[22] Kelly et al. found no significant association between baseline pre-frailty or baseline frailty and subsequent mortality,[23] so we assumed hazard ratios of 1 for the analyses based on their findings. The top section of the table shows per-person values, and the bottom section shows the population total scaling the per-person results to the estimated population size (521,994 people).

© 2026 Smith KC et al. *JAMA Network Open*.

eTable 13. Burden of Prefrailty, Frailty, and Falls Among People With HIV and Viral Suppression Relative to Scenarios With Intervention-Based Reductions in Prefrailty, Frailty, and Falls

	Per Person (95% UI)		
**Attributable to**	**Life-Years Lost**	**QALYs Lost**	**Attributable Costs**
Pre-Frailty and Frailty	1.00 (0.06 – 2.67)	0.85 (0.15 – 2.07)	$7,800 ($2,600 – $13,500)
Falls	0.04 (0.01 – 0.07)	0.03 (0.01 – 0.05)	$700 ($200 – $1,300)
	**Population Total (95% UI)**		
**Attributable to**	**Life-Years Lost**	**QALYs Lost**	**Attributable Costs**
Pre-Frailty and Frailty	522,000 (31,000 – 1,394,000)	444,000 (78,000 – 1,081,000)	$4.1B $1.4B – $7.0B)
Falls	21,000 (5,000 – 37,000)	16,000 (5,000 – 26,000)	$365M ($104M – $679M)

Abbreviations: UI, uncertainty interval; QALYs, quality-adjusted life-years; B, billions; M, millions.

The table shows life-years lost, QALYs lost, and lifetime costs attributable to pre-frailty, frailty, and falls comparing the status quo scenario to a scenario with a reduction in fall or frailty risk estimated from fall and frailty prevention interventions. The top section of the table shows per-person values, and the bottom section shows the population total scaling the per-person results to the estimated population size (521,994 people).

© 2026 Smith KC et al. *JAMA Network Open*.

eTable 14. Burden of Prefrailty, Frailty, and Falls Among People With HIV and Viral Suppression Relative to People Without HIV

	Per Person (95% UI)		
**Attributable to**	**Life-Years Lost**	**QALYs Lost**	**Attributable Costs**
Pre-Frailty and Frailty	0.06<br>(0.00 – 0.13)	0.05<br>(0.01 – 0.10)	$700<br>($500 – $1,000)
Falls	0.07<br>(0.04 – 0.11)	0.05<br>(0.03 – 0.07)	$700<br>($300 – $1,200)
	**Population Total (95% UI)**		
**Attributable to**	**Life-Years Lost**	**QALYs Lost**	**Attributable Costs**
Pre-Frailty and Frailty	31,000<br>(0 – 68,000)	26,000<br>(5,000 – 52,000)	$365M<br>($261M – $522M)
Falls	37,000<br>(21,000 – 57,000)	26,000<br>(16,000 – 37,000)	$365M<br>($157M – $626M)

Abbreviations: UI, uncertainty interval; QALYs, quality-adjusted life-years; M, millions.

The table shows life-years lost, QALYs lost, and lifetime costs attributable to pre-frailty, frailty, and falls comparing the status quo scenario to a scenario based on frailty and fall prevalence among people without HIV. The top section of the table shows per-person values, and the bottom section shows the population total scaling the per-person results to the estimated population size (521,994 people).

© 2026 Smith KC et al. *JAMA Network Open*.

eFigure 1. Model Validation for Frailty Status

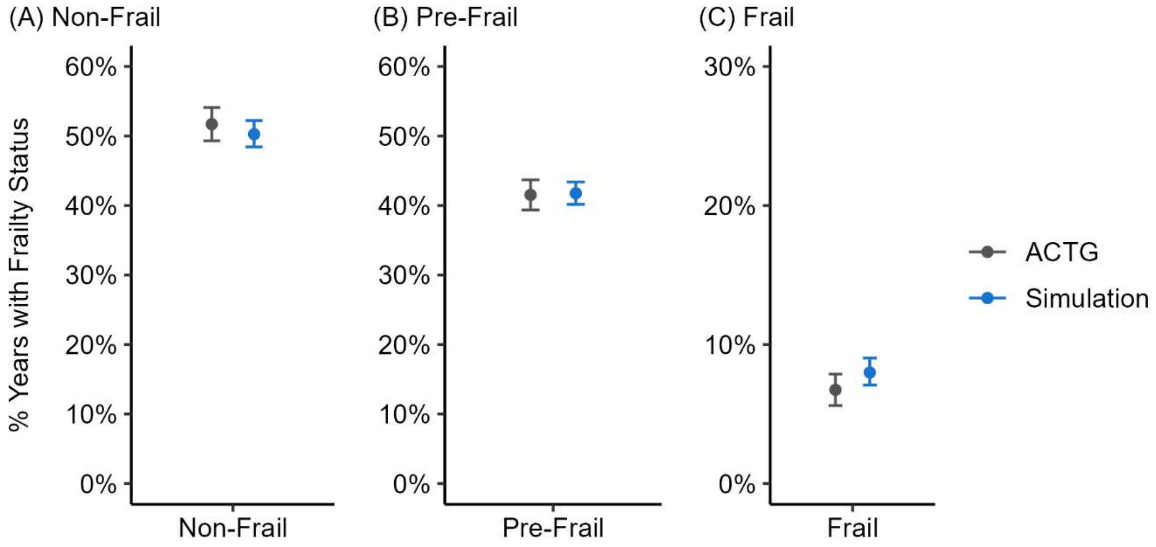

The figure shows the percentage of annual visits with each reported frailty status in the ACTG A5322 study (in black) compared to the percentage of years the simulated A5322 cohort spent in each frailty state (in blue). Error bars represent 95% confidence intervals for the ACTG A5322 results and 95% uncertainty intervals for the simulation results.

© 2026 Smith KC et al. *JAMA Network Open*.

eFigure 2. Model Validation for Fall-Related Events

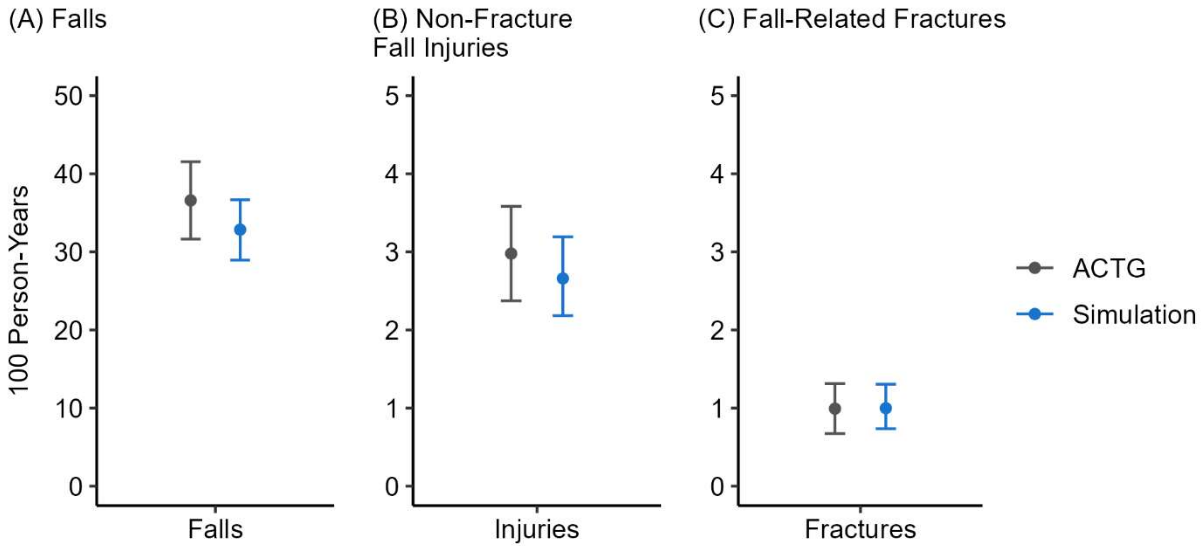

The figure shows the rate of falls, non-fracture fall-injuries, and fall related fractures in the ACTG A5322 study (in black) compared to the rates in the simulation of the A5322 cohort (in blue). Error bars represent 95% confidence intervals for the ACTG A5322 results and 95% uncertainty intervals for the simulation results.

© 2026 Smith KC et al. *JAMA Network Open*.

eFigure 3. External Validation of 2-Year Frailty Trajectories

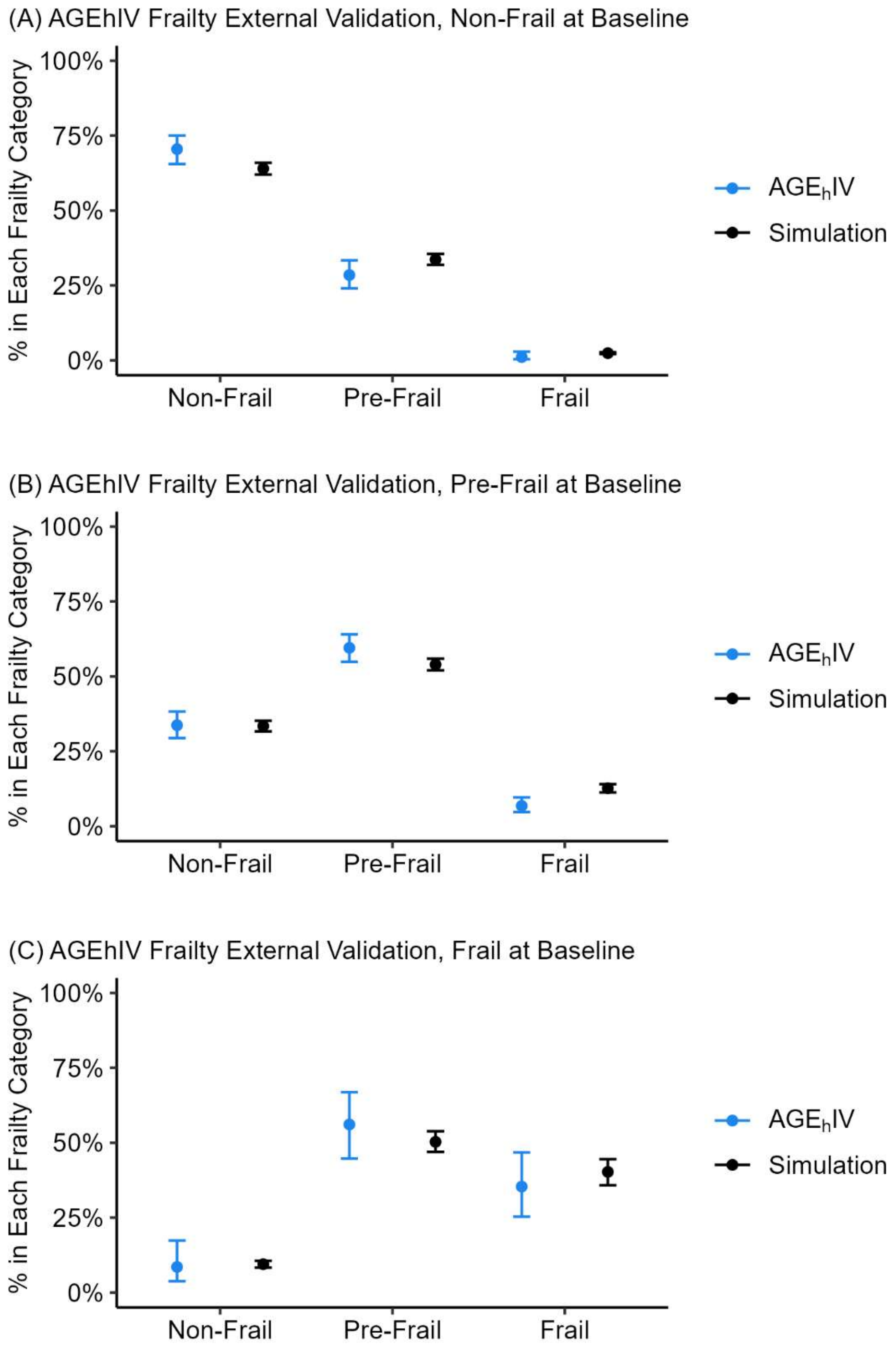

The figure shows the percentage of people in each frailty state after two years for those starting the in (A) the non-frail state, (B) the pre-frail state, and (C) the frail state. The blue AGE_hIV values on the left are based on estimates reported by Verheij and colleagues for two-year frailty transitions among people with HIV in the AGE_hIV cohort.[30] Error bars are 95% confidence intervals for the AGE_hIV outcomes and 95% uncertainty intervals for simulated outcomes.

© 2026 Smith KC et al. *JAMA Network Open*.

eFigure 4. External Validation of 2-Year Fall Incidence

(A) MACS Bone Strength Substudy External Validation

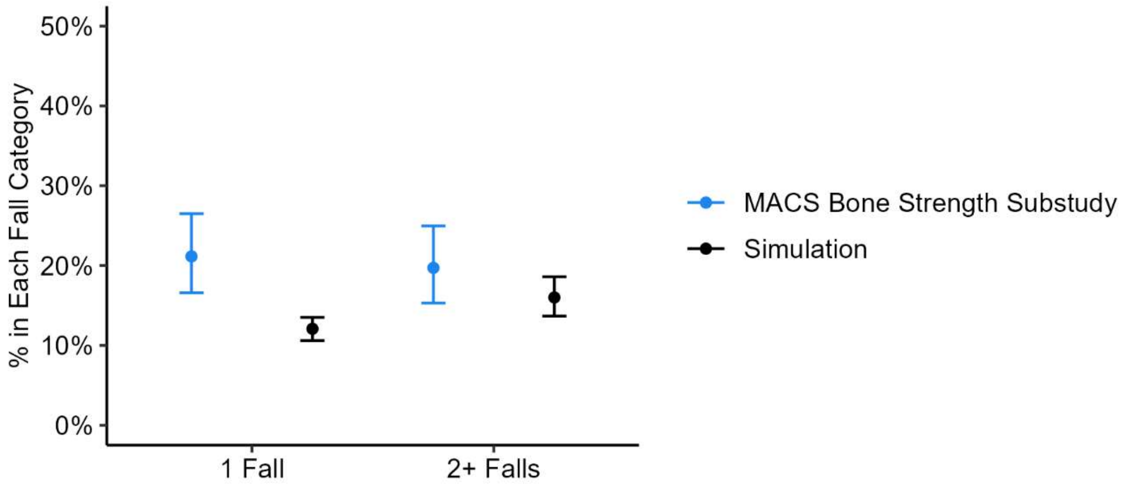

(B) WIHS External Validation

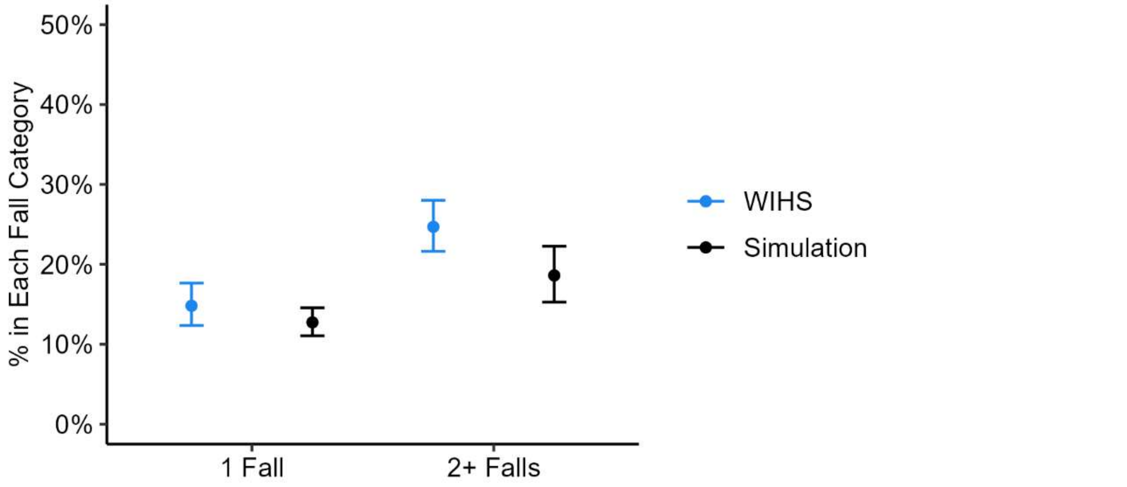

The figure shows the percentage reporting one or two or more falls over two years in (A) the MACS Bone Strength Substudy, reported by Erlandson and colleagues[31] and (B) WIHS 2014-2016 study visits, reported by Sharma and colleagues.[32] Error bars are 95% confidence intervals for the observed outcomes and 95% uncertainty intervals for simulated outcomes.

© 2026 Smith KC et al. *JAMA Network Open*.

eFigure 5. Projected Frailty and Fall Status by Year of Age

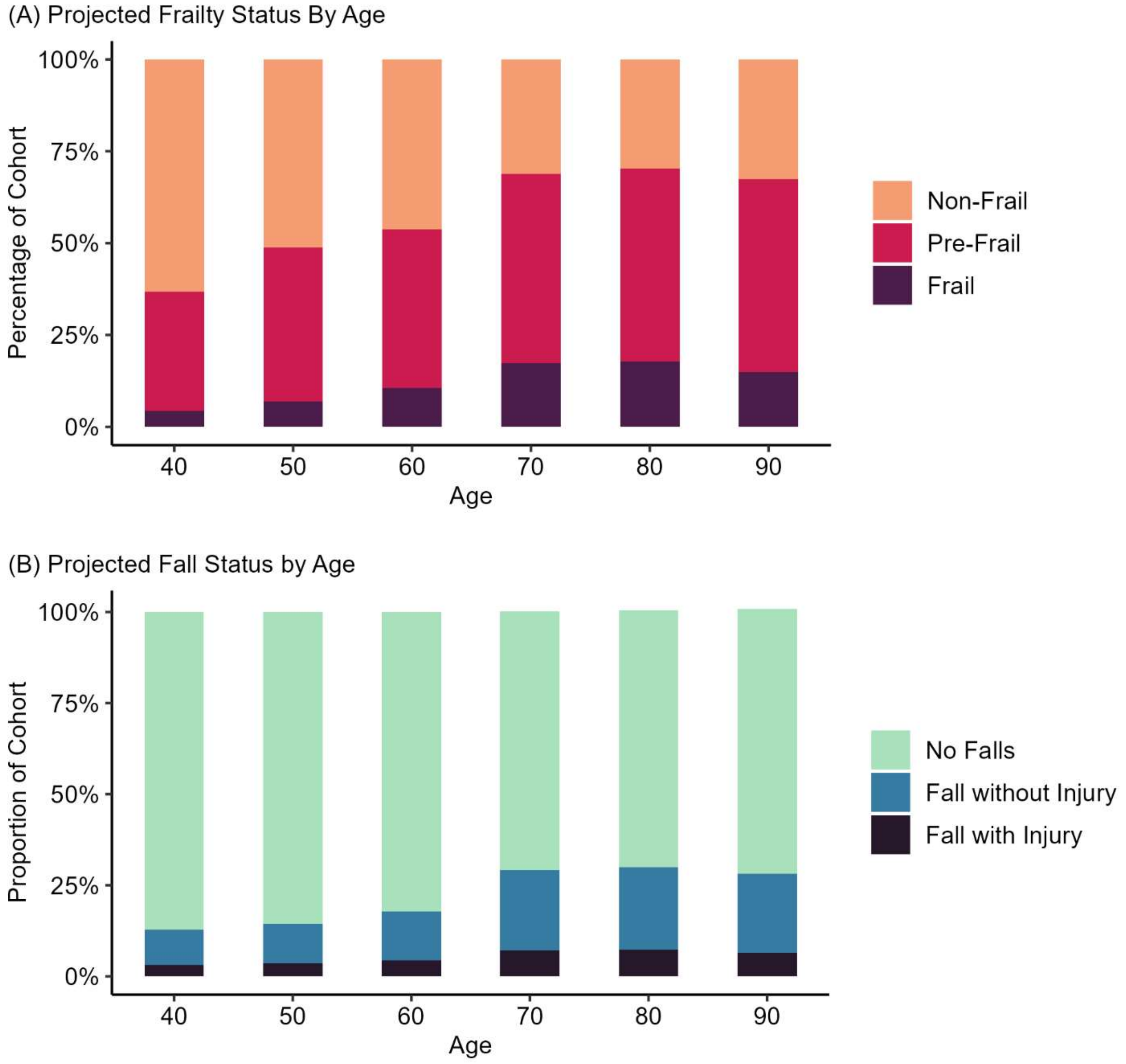

The figure shows the percentage of simulated individuals who are in each frailty or fall state out of those who are alive using results from a simulation starting all individuals at age 40 (100% alive at age 40, 92% at age 50, 80% at age 60, 61% at age 70, 34% at age 80, and 8% at age 90).

© 2026 Smith KC et al. *JAMA Network Open*.

eFigure 6. Age-Stratified Life-Years Lost, QALYs Lost, and Attributable Costs

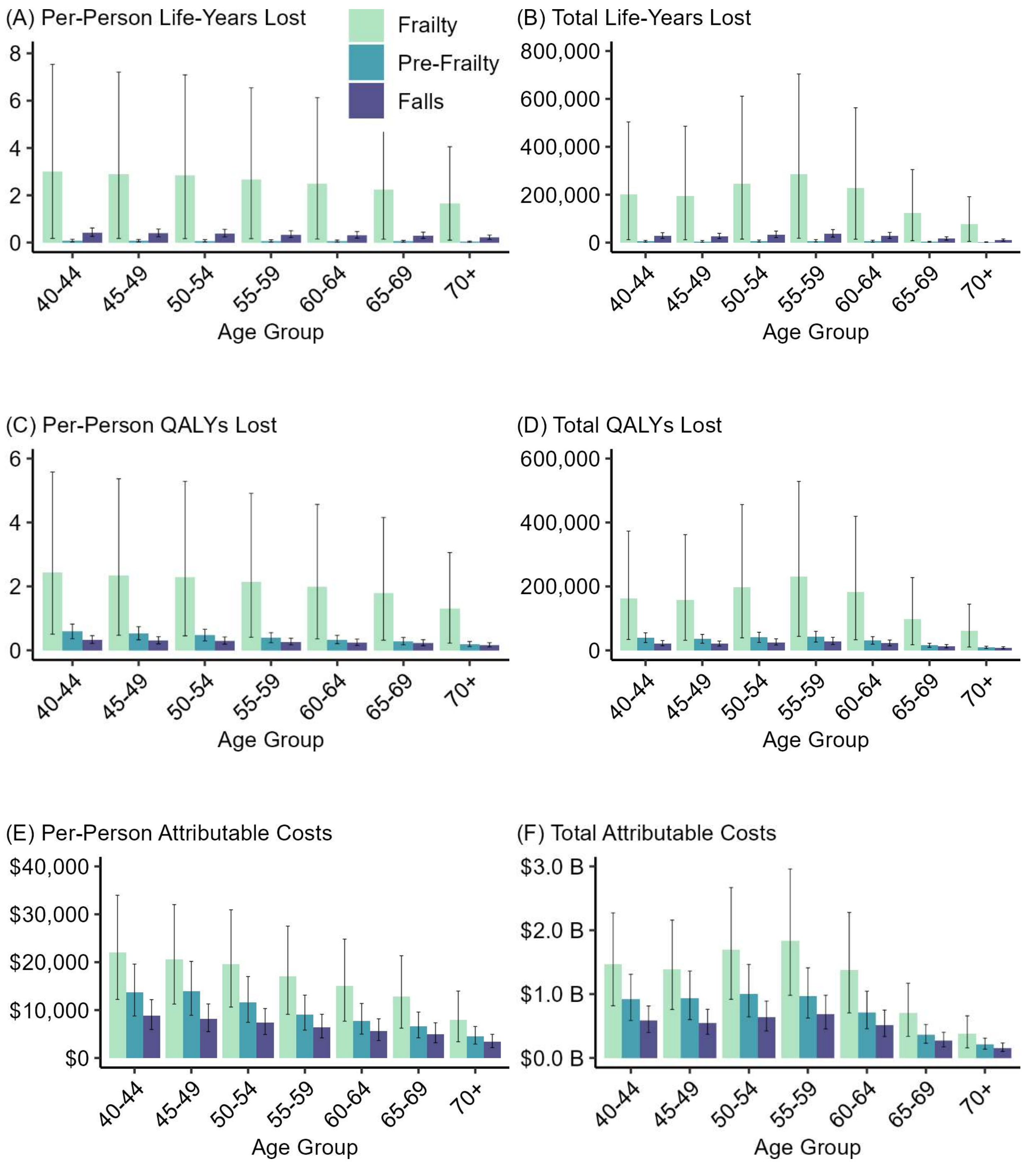

Model results are shown by age group using each simulated individual's age at the start of the simulation. Panels A, C, and E show per-person level outcomes, while panels B, D, and F show outcomes at the population level for the estimated 521,994 people with HIV and viral suppression in the United States aged 40 and over in 2022. eTable 8 provides the remaining life expectancy for each age group. Abbreviations: QALYs, quality-adjusted life-years.

© 2026 Smith KC et al. *JAMA Network Open*.

eReferences

1. Krijkamp EM, Alarid-Escudero F, Enns EA, Jalal HJ, Hunink MGM, Pechlivanoglou P. Microsimulation modeling for health decision sciences using R: A tutorial. *Med Decis Making*. Apr 2018;38(3):400-422. doi:10.1177/0272989X18754513

2. Jalal H, Pechlivanoglou P, Krijkamp E, Alarid-Escudero F, Enns E, Hunink MGM. An overview of R in health decision sciences. *Med Decis Making*. Oct 2017;37(7):735-746. doi:10.1177/0272989X16686559

3. A5322: Long-Term Follow-up of Older HIV-infected Adults in the ACTG: Addressing Issues of Aging, HIV Infection and Inflammation (HAILO). https://actgnetwork.org/clinical-trial/a5322-long-term-follow-up-of-older-hiv-infected-adults-in-the-actg-addressing-issues-of-aging-hiv-infection-and-inflammation-hailo-2/. Date Accessed: November 15, 2024.

4. Multicenter AIDS Cohort Study (MACS) / Women's Interagency HIV Study (WIHS) Combined Cohort Study. https://www.nhlbi.nih.gov/science/macswihs-combined-cohort-study. Date Accessed: November 15, 2024.

5. Centers for Disease Control and Prevention. Diagnoses, deaths, and prevalence of HIV in the United States and 6 territories and freely associated states, 2022. *HIV Surveillance Report, 2022; vol 35*. http://www.cdc.gov/hiv-data/nhss/hiv-diagnoses-deaths-prevalence.html. Published May 2024. Date Accessed: December 30, 2024.

6. Centers for Disease Control and Prevention (CDC). Monitoring selected national HIV prevention and care objectives by using HIV surveillance data—United States and 6 territories and freely associated states, 2022. *HIV Surveillance Supplemental Report 2024;29(No 2)*. https://www.cdc.gov/hiv-data/nhss/national-hiv-prevention-and-care-outcomes.html. Published May 2024. Date Accessed: December 30, 2024.

7. Achour J, Abulizi D, Makinson A, et al. One-year frailty transitions among persons with HIV aged 70 years or older on antiretroviral treatment. *Open Forum Infect Dis*. Jul 2024;11(7):ofae229. doi:10.1093/ofid/ofae229

8. Shebl FM, Qian Y, Foote JHA, et al. The association between all-cause mortality and HIV acquisition risk groups in the United States, 2001-2014. *PLoS One*. 2023;18(8):e0290113. doi:10.1371/journal.pone.0290113

9. Verheij E, Kirk GD, Wit FW, et al. Frailty is associated with mortality and incident comorbidity among middle-aged human immunodeficiency virus (HIV)-positive and HIV-negative participants. *J Infect Dis*. Aug 17 2020;222(6):919-928. doi:10.1093/infdis/jiaa010

10. Tosteson AN, Gottlieb DJ, Radley DC, Fisher ES, Melton LJ, 3rd. Excess mortality following hip fracture: the role of underlying health status. *Osteoporos Int*. Nov 2007;18(11):1463-72. doi:10.1007/s00198-007-0429-6

11. Centers for Medicare & Medicaid Services. National Health Expenditure Accounts (NHEA): Personal Health Care (PCE) Expenditure Deflator. Accessed January 27, 2025,

https://www.cms.gov/Research-Statistics-Data-and-Systems/Statistics-Trends-and-Reports/NationalHealthExpendData/NationalHealthAccountsHistorical

12. Ensrud KE, Schousboe JT, Kats AM, Taylor BC, Boyd CM, Langsetmo L. Incremental health care costs of self-reported functional impairments and phenotypic frailty in community-dwelling older adults : A prospective cohort study. *Ann Intern Med*. Apr 2023;176(4):463-471. doi:10.7326/M22-2626

13. Hansen D, Pelizzari PM, Pyenson BS. Medicare cost of osteoporotic fractures: 2021 updated report. *Milliman Research Report*.

14. Choi NG, Choi BY, DiNitto DM, Marti CN, Kunik ME. Fall-related emergency department visits and hospitalizations among community-dwelling older adults: Examination of health problems and injury characteristics. *BMC Geriatr*. Nov 11 2019;19(1):303. doi:10.1186/s12877-019-1329-2

15. Peterson C, Xu L, Florence C. Average medical cost of fatal and non-fatal injuries by type in the USA. *Inj Prev*. Feb 2021;27(1):24-33. doi:10.1136/injuryprev-2019-043544

16. Haddad YK, Shakya I, Moreland BL, Kakara R, Bergen G. Injury diagnosis and affected body part for nonfatal fall-related injuries in community-dwelling older adults treated in emergency departments. *J Aging Health*. Dec 2020;32(10):1433-1442. doi:10.1177/0898264320932045

17. Wang B, Mehrotra A, Friedman AB. Urgent care centers deter some emergency department visits but, on net, increase spending. *Health Aff (Millwood)*. Apr 2021;40(4):587-595. doi:10.1377/hlthaff.2020.01869

18. Figueroa JF, Katz IT, Hyle EP, et al. The association of HIV with health care spending and use among Medicare beneficiaries. *Health Aff (Millwood)*. Apr 2022;41(4):581-588. doi:10.1377/hlthaff.2021.01793

19. Bozzette SA, Hays RD, Berry SH, Kanouse DE, Wu AW. Derivation and properties of a brief health status assessment instrument for use in HIV disease. *J Acquir Immune Defic Syndr Hum Retrovirol*. Mar 1 1995;8(3):253-65. doi:10.1097/00042560-199503010-00006

20. Brazier J, Roberts J, Deverill M. The estimation of a preference-based measure of health from the SF-36. *J Health Econ*. Mar 2002;21(2):271-92. doi:10.1016/s0167-6296(01)00130-8

21. Raich W, Baxter J, Sheahan M, Goldhaber-Fiebert J, Sullivan P, Hanmer J. Estimates of quality-adjusted life-year loss for injuries in the United States. *Med Decis Making*. Apr 2023;43(3):288-298. doi:10.1177/0272989X221141454

22. Akgun KM, Tate JP, Crothers K, et al. An adapted frailty-related phenotype and the VACS index as predictors of hospitalization and mortality in HIV-infected and uninfected individuals. *J Acquir Immune Defic Syndr*. Dec 1 2014;67(4):397-404. doi:10.1097/QAI.0000000000000341

© 2026 Smith KC et al. *JAMA Network Open*.

23. Kelly SG, Wu K, Tassiopoulos K, Erlandson KM, Koletar SL, Palella FJ. Frailty is an independent risk factor for mortality, cardiovascular disease, bone disease, and diabetes among aging adults with human immunodeficiency virus. *Clin Infect Dis*. Sep 27 2019;69(8):1370-1376. doi:10.1093/cid/ciy1101

24. Macdonald SH, Travers J, She EN, et al. Primary care interventions to address physical frailty among community-dwelling adults aged 60 years or older: A meta-analysis. *PLoS One*. 2020;15(2):e0228821. doi:10.1371/journal.pone.0228821

25. Guirguis-Blake JM, Perdue LA, Coppola EL, Bean SI. Interventions to prevent falls in older adults: Updated evidence report and systematic review for the US Preventive Services Task Force. *JAMA*. Jul 2 2024;332(1):58-69. doi:10.1001/jama.2024.4166

26. Bandeen-Roche K, Seplaki CL, Huang J, et al. Frailty in older adults: A nationally representative profile in the United States. *J Gerontol A Biol Sci Med Sci*. Nov 2015;70(11):1427-34. doi:10.1093/gerona/glv133

27. Hanlon P, Nicholl BI, Jani BD, Lee D, McQueenie R, Mair FS. Frailty and pre-frailty in middle-aged and older adults and its association with multimorbidity and mortality: a prospective analysis of 493 737 UK Biobank participants. *Lancet Public Health*. Jul 2018;3(7):e323-e332. doi:10.1016/S2468-2667(18)30091-4

28. Kakara R, Bergen G, Burns E, Stevens M. Nonfatal and fatal falls among adults aged ≥65 Years - United States, 2020-2021. *MMWR Morb Mortal Wkly Rep*. Sep 1 2023;72(35):938-943. doi:10.15585/mmwr.mm7235a1

29. Verma SK, Willetts JL, Corns HL, Marucci-Wellman HR, Lombardi DA, Courtney TK. Falls and fall-related injuries among community-dwelling adults in the United States. *PLoS One*. 2016;11(3):e0150939. doi:10.1371/journal.pone.0150939

30. Verheij E, Wit FW, Verboeket SO, et al. Frequency, risk factors, and mediators of frailty transitions during long-term follow-up among people with HIV and HIV-negative AGEhIV cohort participants. *J Acquir Immune Defic Syndr*. Jan 1 2021;86(1):110-118. doi:10.1097/QAI.0000000000002532

31. Erlandson KM, Zhang L, Ng DK, et al. Risk factors for falls, falls with injury, and falls with fracture among older men with or at risk of HIV infection. *J Acquir Immune Defic Syndr*. Aug 1 2019;81(4):e117-e126. doi:10.1097/QAI.0000000000002074

32. Sharma A, Hoover DR, Shi Q, et al. Frailty as a predictor of falls in HIV-infected and uninfected women. *Antivir Ther*. 2019;24(1):51-61. doi:10.3851/IMP3286

© 2026 Smith KC et al. *JAMA Network Open*.
