# Supplementary material for: Modeled Health and Economic Burden of Frailty and Falls Among Adults With HIV
Source: JAMA Netw Open. 2026 Jan 21;9(1):e2554809. doi: 10.1001/jamanetworkopen.2025.54809 (PMC12824775; doi:10.1001/jamanetworkopen.2025.54809)
Supplement: Supplement 2. — Data Sharing Statement [file jamanetwopen-e2554809-s002.pdf]

Data Sharing Statement

Smith. Modeled Health and Economic Burden of Frailty and Falls Among Adults With HIV. *JAMA Netw Open*. Published January 21, 2026. doi:10.1001/jamanetworkopen.2025.54809

Data
Data available: No

Additional Information
Explanation for why data not available: Individual-level patient data were not collected for this study. Additional information on model parameters is available via request to Dr. Karen Smith (ksmith81@bwh.harvard.edu).
